# Characterization of CADD522, a small molecule that inhibits RUNX2-DNA binding and exhibits antitumor activity

**DOI:** 10.18632/oncotarget.20200

**Published:** 2017-08-10

**Authors:** Myoung Sook Kim, Ramkishore Gernapudi, Eun Yong Choi, Rena G. Lapidus, Antonino Passaniti

**Affiliations:** ^1^ Department of Pathology, University of Maryland School of Medicine, Baltimore, MD, USA; ^2^ Department of Biochemistry & Molecular Biology and Program in Molecular Medicine, University of Maryland School of Medicine, Baltimore, MD, USA; ^3^ The Marlene & Stewart Greenebaum Comprehensive Cancer Center, University of Maryland School of Medicine, Baltimore, MD, USA; ^4^ The Veteran’s Health Administration Research & Development Service, Baltimore, MD, USA

**Keywords:** breast cancer, RUNX2, therapeutics

## Abstract

The RUNX2 transcription factor promotes breast cancer growth and metastasis through interactions with a variety of cofactors that activate or repress target genes. Using a direct drug discovery approach we identified CADD522 as a small molecule that inhibits the DNA binding of the runt box domain protein, RUNX2. The current study defines the effect of CADD522 on breast cancer growth and metastasis, and addresses the mechanisms by which it exerts its anti-tumor activity.

CADD522 treatment resulted in significant growth inhibition, clonogenic survival, tumorsphere formation, and invasion of breast cancer cells. CADD522 negatively regulated transcription of RUNX2 target genes such as matrix metalloproteinase-13, vascular endothelial growth factor and glucose transporter-1, but upregulated RUNX2 expression by increasing RUNX2 stability. CADD522 reduced RUNX2-mediated increases in glucose uptake and decreased the level of CBF-β and RUNX2 phosphorylation at the S451 residue. These results suggest several potential mechanisms by which CADD522 exerts an inhibitory function on RUNX2-DNA binding; interference with RUNX2 for the DNA binding pocket, inhibition of glucose uptake leading to cell cycle arrest, down-regulation of CBF-β, and reduction of S451-RUNX2 phosphorylation.

The administration of CADD522 into MMTV-PyMT mice resulted in significant delay in tumor incidence and reduction in tumor burden. A significant decrease of tumor volume was also observed in a CADD522-treated human triple-negative breast cancer-patient derived xenograft model. CADD522 impaired the lung retention and outgrowth of breast cancer cells *in vivo* with no apparent toxicity to the mice. Therefore, by inhibiting RUNX2-DNA binding, CADD522 may represent a potential antitumor drug.

## INTRODUCTION

Despite recent advances in treatment, breast cancer (BC) still remains the second leading cause of cancer-related deaths among women [1]. Luminal BC has the highest rates of relapse, often localizes to the bone or lung [2, 3] and accounts for 50% of all metastasis-related BC deaths in spite of the primary tumor being highly responsive to treatment [4]. Given their high rate of relapse, it is clear that current treatment modalities are insufficient to completely eradicate these heterogeneous tumors. Triple-negative breast cancer (TNBC) accounts for 15% ∼ 20% of all breast cancer cases [5-7] and is characterized by poor survival and a high probability of early treatment relapse, especially distant metastasis [8-10]. Because of no useful biomarkers and no targeted therapeutic modalities for metastatic disease, treatment options for patients with TNBC are limited to cytotoxic chemotherapy. Thus, there is an urgent need to elucidate novel targets for BC therapy to improve the poor prognosis for patients.

The runt-related transcription factor-2 (RUNX2) promotes BC progression and metastasis through transcriptional activation of its target genes [11-13]. RUNX2 serves as a convergence node for different signaling pathways [14]. For both luminal and triple-negative BC, RUNX2 expression is associated with poor clinical prognosis [15, 16]. RUNX2 interacts with its specific DNA recognition site on gene promoters through its Runt domain that consists of 128 amino acids, highly conserved and exclusively found in the RUNX proteins (RUNX1, 2, and 3). RUNX2 is a specific transcription factor that mediates complex metabolic events such as angiogenesis or glycolysis by promoting glycolytic switching or by inhibiting mitochondrial oxidative phosphorylation [17, 18]. RUNX2 decreases PDH activity, but its knockdown increases oxygen consumption rate (OCR) [17] by increasing the activity of pyruvate dehydrogenase (PDH), a rate limiting step for entry into the TCA cycle at the branch point for pyruvate utilization. These data imply that targeting RUNX2 might inhibit BC growth and/or progression by reversing tumor cell dependence on glycolysis. RUNX1 plays fundamental roles in definitive hematopoiesis, and genetic aberrations of RUNX1 gene are involved in the pathogenesis of leukemia [19, 20]. Emerging evidence suggests that RUNX1 is relevant for BC promotion and metastasis. Recent whole-genome and whole-exome sequencing studies have revealed that RUNX1 mutations are common in BC [21, 22], and in particular, some point mutations in RUNX1 DNA-binding domain abolish its DNA binding activity [21, 22], while RUNX2 mutations are rare [23]. The expression of RUNX1 is associated with BC progression [24], and correlates with poor prognosis in TNBC patients [25]. RUNX3 silencing correlates with progression of gastric cancer [26-29], and frequently inactivated in BC [30, 31].

Recent advances in determination of three-dimensional architecture and dynamics of transcription factor complexes in cancer cells have promoted development of new types of anticancer agents with excellent potential for clinical applications [32, 33]. Transcription Therapy is an emerging approach that reverses aberrant gene expression in cancer cells through direct or indirect targeting of the transcriptional process [34, 35]. This type of drug development could restore homeostasis in cellular gene expression and inhibit tumor progression. Due to redundancies in normal signaling pathways [35], modulating the activity of transcription factors might exhibit low toxicity with minimal side effects as normal cells often tolerate loss of transcription factor function with little consequence [35]. For example, Nelson et al. identified drugs known to be well tolerated in humans such as specific STAT3 inhibitors, including nifuroxazide (antidiarrhea), pyrimethamine (antimalaria), and pimozide (neurolepsis) by cell-based screens of chemical library of drugs [36-38]. In addition, direct inhibition of STAT3-DNA binding via RNA interference or with oligodeoxynucleotide is progressing into clinical trials with safer and more effective delivery systems [35]. Notch1-selective gamma-secretase inhibitors overcome the gastrointestinal toxicity associated with pan-inhibition of gamma-secretase activity [39]. Although challenges remain in the development of therapeutic agents targeting transcription at the single gene level, it is possible to identify compounds that can alter expression of target genes but have little effect on other transcriptionally co-regulated genes [40].

Targeting RUNX2 can be a highly effective and useful strategy for BC treatment: this approach may exhibit less side effects than conventional chemotherapy and has the potential for high therapeutic index because RUNX2 is normally not expressed in differentiated bone or mature glandular tissues, such as breast [17, 41] and prostate [42]. In spite of these benefits, therapeutic agents are very rare. We have taken a direct drug discovery approach using Computer-Assisted Drug Design (CADD) [43, 44] to find novel compounds that interact with RUNX2 and DNA by fitting into the active Runt-DNA binding pocket [43, 44]. CADD522 was one of the compounds identified as a potent inhibitor of RUNX2-DNA binding [43, 44]. Using DNA-binding ELISA (D-ELISA) with a RUNX2-specific antibody, we validated that CADD522 inhibited *in vitro* DNA binding activity of RUNX2 at nanomolar concentrations (IC50 @ 10 nM) [43, 44]. Interestingly, CADD522 inhibited tumorsphere formation of luminal BC cells expressing ectopic RUNX2 [16], supporting the therapeutic potential of CADD522 as an anticancer drug for BC therapy.

Herein, we report that CADD522, a small molecule inhibitor of RUNX2, downregulates RUNX2-mediated transcription of downstream target genes by inhibiting RUNX2-DNA binding. *In vitro* and *in vivo* studies reveal that CADD522 suppresses BC growth and metastasis. Our observations suggest that CADD522 has therapeutic potential to restrict breast cancer progression.

## RESULTS

### CADD522 suppresses *in vitro* BC cell growth and survival

From our CADD screening [43, 44], CADD522 was identified as a novel inhibitor of RUNX2-DNA binding (Supplementary Figure 1A). To assess the relative specificity of the CADD522 compound for RUNX family proteins, we performed D-ELISA with osteoblast specific element 2 (OSE2) oligonucleotides from the RUNX2-regulated Osteocalcin (OC) gene promoter and specific RUNX1, RUNX2 or RUNX3 antibodies [43, 44]. CADD522 inhibited the DNA binding activity of all RUNX proteins, but most prominent inhibition was observed for RUNX2-DNA binding (Supplementary Figure 1B). MMP13 is a well-known RUNX2 target gene [45, 46] that harbors a Runt binding site (AACCACA) in the promoter at -160 bp from the start codon. We performed D-ELISA with MMP13 oligonucleotides designed from the promoter sequences near the Runt binding site, and found that CADD522 also inhibited RUNX2 binding to the MMP13 oligonucleotides in a dose-dependent manner (Supplementary Figure 1C). Consistently, ChIP analysis showed increased enrichment of RUNX2 on MMP13 promoters in the MCF7-RUNX2 cells compared to the MCF7-Empty control (Supplementary Figure 1D). While CADD522 had no effect on the RUNX2 occupancy in MCF7-Empty cells, CADD522 significantly inhibited RUNX2 enrichment in the MCF7-RUNX2 cells (Supplementary Figure 1D). These results indicate that CADD522 inhibits RUNX2-DNA binding both *in vitro* and *in vivo*.

**Figure 1:**
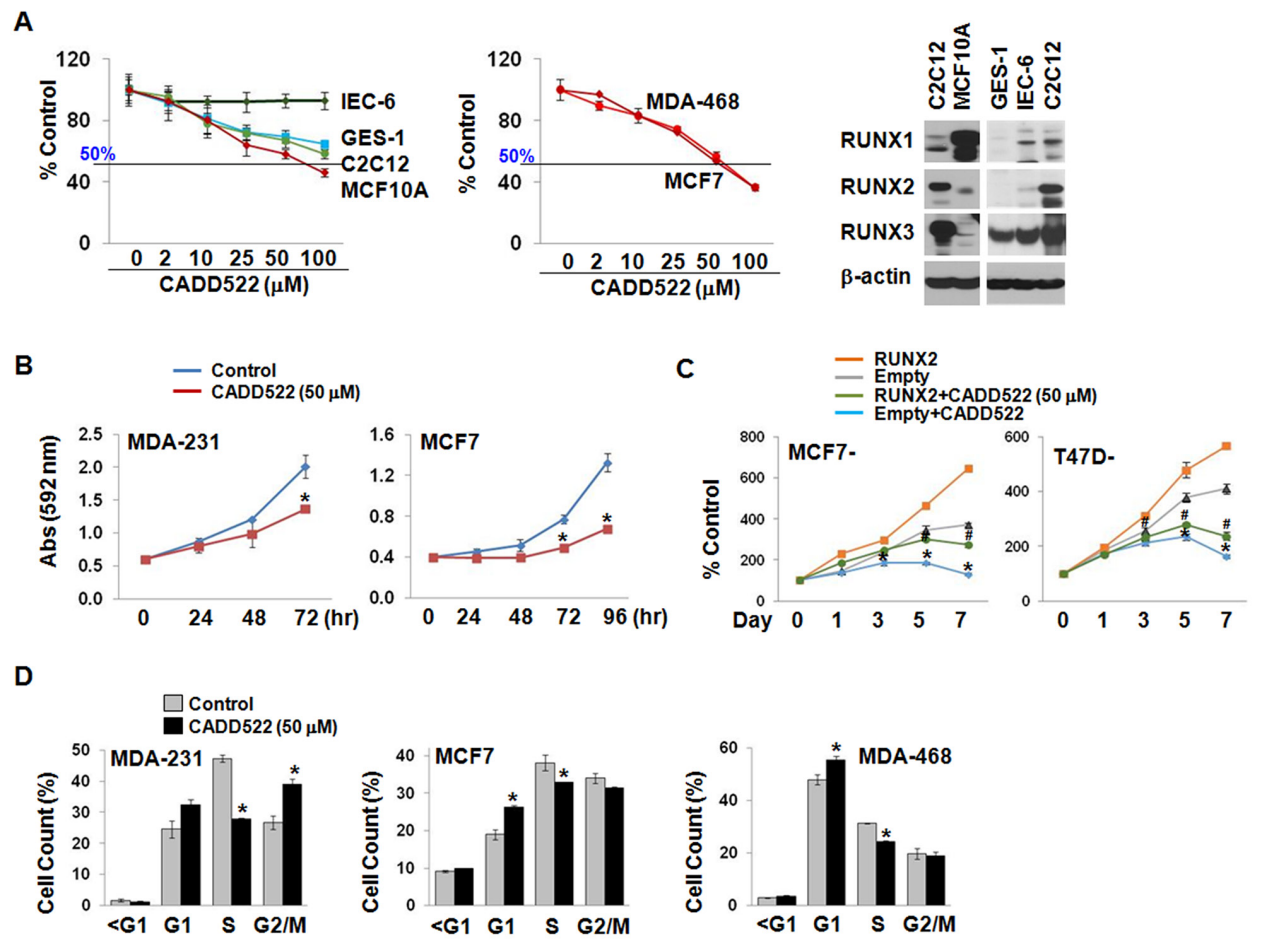
CADD522 suppresses *in vitro* BC cell growth **(A)** Cell growth assay in non-tumorigenic (left) and BC cell lines (middle). Cells were treated with CADD522 for 72 hrs, and cell growth was determined by crystal violet staining. Data presented as mean ± SD. Experiments were done in triplicate and repeated twice. Expression of RUNX proteins in non-tumorigenic cells was determined by western blot analysis (right). **(B)** Time-dependent decrease of MDA-231 and MCF7 cell growth by CADD522. *, *P<0.05* compared to vehicle control at indicated time. **(C)** Time-dependent decrease of ectopic RUNX2-expressing MCF7 and T47D cells compared to Empty controls. cell growth was calculated as percentage (%) absorbance at indicated time point relative to absorbance of cells at Day 0. *, *P<0.05* compared to Empty controls treated with vehicle alone (0.1% DMSO). ^#^, *P<0.05* compared to RUNX2-exprssing cells with vehicle alone. * and ^#^, *P<0.05* considered significant. **(D)** Cell population at each phase of the cell cycle was analyzed by flow cytometry. MDA-231 cells accumulated at the G1 and G2/M phase whereas MCF7 and MDA-468 cells were at the G1 phase after CADD522 treatment.

Targeting Runt-DNA binding may be selective for BC cells as RUNX2 is not expressed in normal mammary epithelial cells [17, 41], but it would be of a concern in normal cells that express RUNX1 and/or RUNX3. To determine the effect of CADD522 on cell viability and growth, we treated BC cells (MDA-MB-468, and MCF7) as well as non-malignant cells (MCF10A untransformed human mammary epithelial cells, IEC-6 undifferentiated rat intestinal cells, and GES-1 human gastric mucosal cells, and C2C12 murine myoblast cells) with CADD522 (0 ∼ 100 μM) for 24 ∼ 72 hrs. There was no significant inhibition in cell viability over 24 hrs (data not shown), while CADD522 displayed a dose- and time-dependent cell growth inhibition over 72 hrs (Figure 1A & 1B). However, the sensitivity of non-malignant cells to CADD522 was much lower than that of BC cell lines, indicating that CADD522 might not exhibit serious cytotoxicity for normal cell growth.

When ectopic RUNX2-expressing MCF7 and T47D cells were treated with CADD522 for 7 days, a dramatic decrease in cell proliferation relative to vehicle controls (Figure 1C) was observed. Next, we explored the growth inhibitory effect of CADD522 on other BC cell lines. As shown in Supplementary Figure 2A, CADD522 at 50 μM for 72 hrs exerted mild but significant growth inhibition (< 50%) in the majority of TNBC and luminal type BC cells. Among them, MDA-MB-468 (MDA-468) cells were most sensitive to CADD522 (> 50%). Moreover, CADD522 at 50 μM within 72 hrs displayed no apoptotic cell death or necrosis under microscopic observations, no increase in the levels of cleaved caspase-3/7 expression (data not shown), and no change in caspase-3/7 activity compared to vehicle controls (Supplementary Figure 2B & 2C). However, CADD522 treatment increased cell populations at the G1 phase with reduction at the S phase (Figure 1D & Supplementary Figure 2D), indicating that the anti-proliferative effect of CADD522 might be associated with cell cycle arrest.

**Figure 2:**
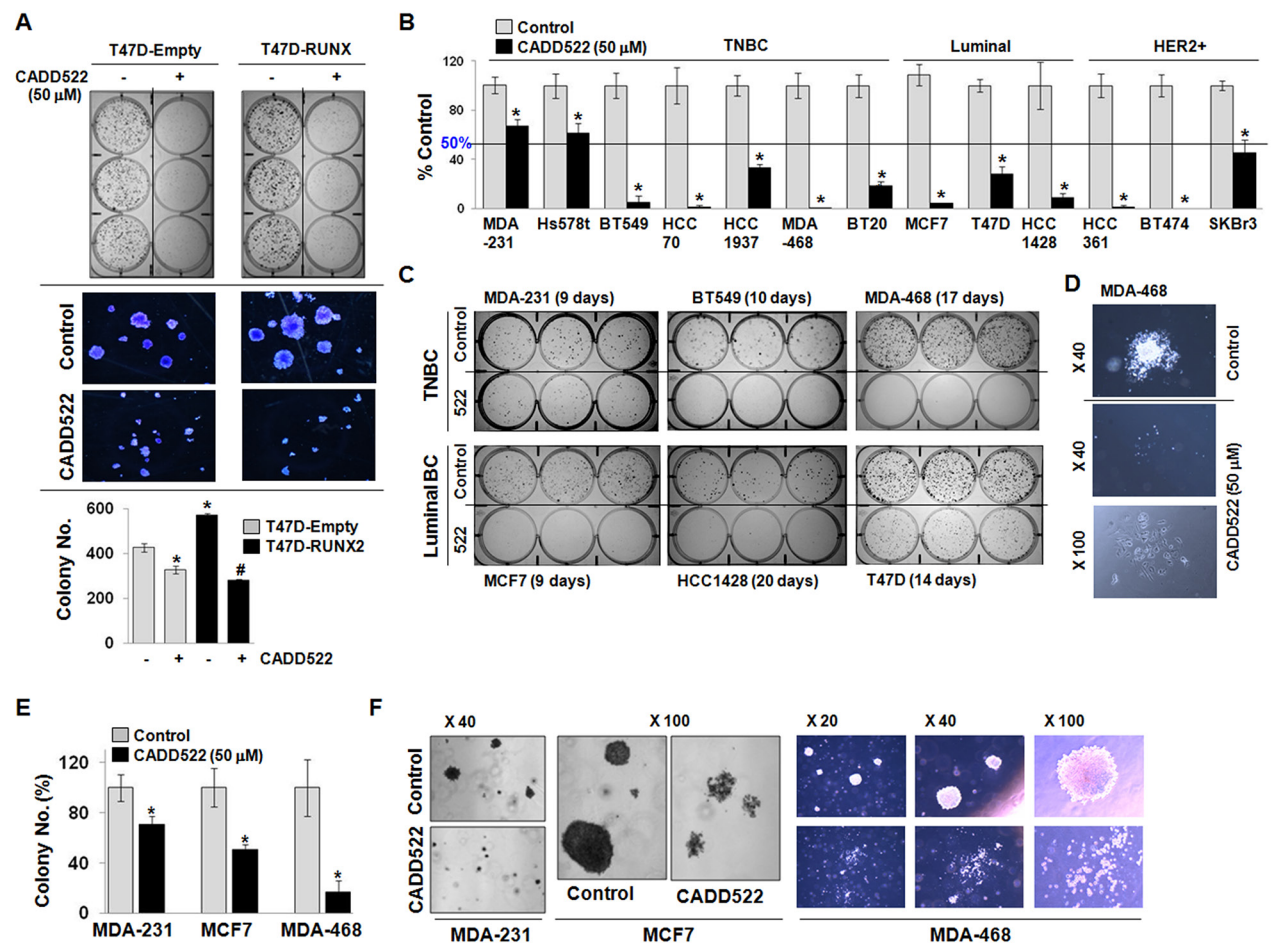
CADD522 diminishes clonogenic survival of BC cells **(A)** T47D-Empty and T47D-RUNX2 cells were treated with CADD522 for 14 days, and clonogenic assay was performed. Photos were taken from colonies in 6-well plate (upper) and under a microscope at X40 magnification (middle), and colonies were counted (lower). *, *P<0.05* compared to the T47D-Empty cells with vehicle alone;^#^, *P<0.05* compared to the T47D-RUNX2 with vehicle alone. -, Vehicle controls; +, CADD522-treated cells. **(B)** Clonogenic survival assay was performed to determine cell survival of BC cell lines after CADD522 treatment (50 μM) for 2 ∼ 3 weeks. Colonies were counted after crystal violet staining. MDA-468 and BT474 did not survive after CADD522 treatment whereas mesenchymal TNBC (MDA-231 and Hs578t) were less sensitive to CADD522 (survival > 50%). Data presented as mean ± SD. Experiments were done in triplicate and repeated twice. *, *P<0.05* compared to control considered significant. **(C)** Colonies were photographed. The periods of CADD522 treatment are indicated for each cell line. **(D)** Photos of single colonies in MDA-468 cells were taken in X40 and X100 magnification. **(E & F)** Anchorage-independent cell growth assays were performed with BC cell lines. Colonies were counted and photographed under a microscope after 2 ∼ 3 weeks of CADD522 treatment.

The effect of CADD522 on long term cell survival was further investigated in BC cells in the presence or absence of CADD522 (50 μM) for 2 ∼ 3 weeks. As shown in Figure 2A, expression of RUNX2 in T47D cells enhanced the growth of colonies (both size and number) compared to Empty vector controls, but CADD522 dramatically diminished the clonogenicity of both T47D-Empty and T47D-RUNX2 cells. The suppression was slightly greater in T47D-RUNX2 cells than in Empty controls. The RUNX2-mediated increase in colony formation was also suppressed by CADD522 in ectopic RUNX2-expressing MCF7 cells (Supplementary Figure 3A). In addition, reduced clonogenicity was observed in CADD522-treated BC cells (11 out of 13 cells) that showed less than 50% survival after CADD522 treatment (Figure 2B & Supplementary Figure 3A). In particular, less than 10% cell survival was observed in six cell lines (BT549, HCC70, MDA-468, MCF7, HCC361, and BT474), indicating that the growth inhibitory effect of CADD522 was not restricted to specific BC cell type. Microscopic images revealed that colonies in the CADD-treated cells were sparse, disintegrated, and smaller than in the vehicle controls that were compact and well-integrated (Figure 2C & 2D). Moreover, CADD522 strongly inhibited anchorage-independent cell growth of BC cells (Figure 2E); colonies of the CADD522-treated MDA-MB-231 (MDA-231), MCF7 and MDA-468 cells were highly disrupted, segregated and apoptotic, whereas those of vehicle controls were compact, tight and healthy (Figure 2F). The size of colonies was also much smaller in the CADD522-treated cells than in the control (<1/10 in size). Similar results were observed for CADD522-treated MCF7-tet-off cells in which CADD522 treatment resulted in reduced colony formation (Supplementary Figure 3B). The colony number also was not significantly different in the MCF7-tet-off (+Doxy) and MCF7-tet-off (-Doxy) cells expressing ectopic RUNX2. Taken together, these results indicate that CADD522 has a strong inhibitory effect on BC cell growth and survival

### CADD522 inhibits tumorsphere formation and *in vitro* invasion of BC cells

**Figure 3:**
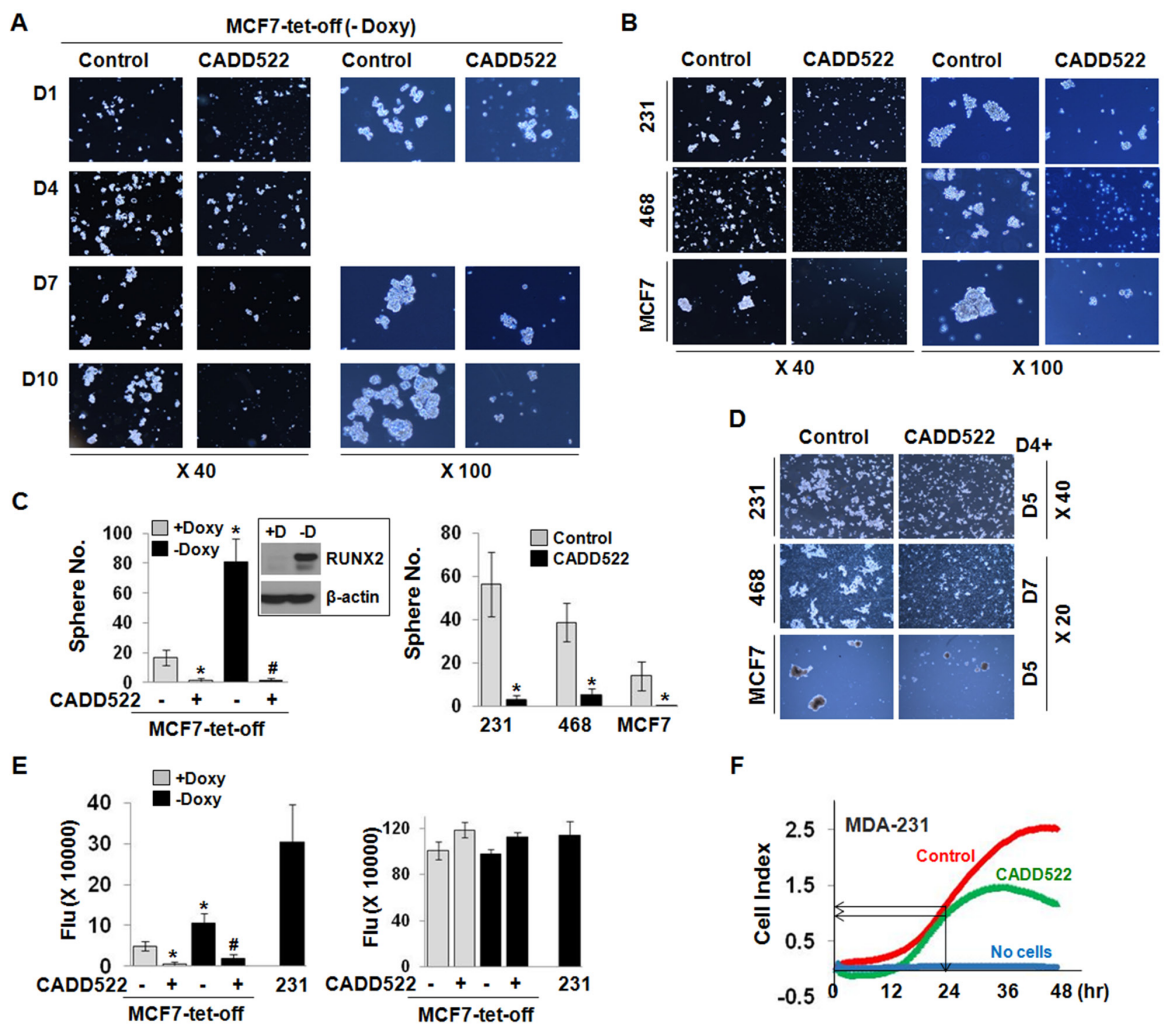
CADD522 inhibits tumorsphere formation and *in vitro* invasion of BC cells **(A-C)** CADD522 was added at the initial day of cell plating. Tumorspheres of MCF7-tet-off (-Doxy) cells were photographed for 18 days at X40 and X100 magnifications (A), and counted at the final day at X40 magnification (C) Sphere No. Photos at day 14 and 18 are shown in Supplementary Figure 4E. Tumorspheres of MDA-231, MDA-468 and MCF7 cells at day 7 (B) Spheres of MDA-231 and MCF7 cells were counted at day 18 with X20 magnification. Spheres of vehicle-treated MDA-468 cells were counted at day 7 with X20 magnification as they became fragile and disrupted 7 days after incubation. Tumorsphere photos of MDA-231, MCF7 and MDA-468 taken at other days are shown in Supplementary Figure 4A - 4C, 4E. 231, MDA-231; 468, MDA-468. (C) Sphere number (No.) was counted from 9 fields per well (described in Materials and Methods). Data presented as mean ± SD. Experiments were done in triplicate and repeated three times. *, P<0.05 compared to +Doxy; ^#^, *P<0.05* compared to -Doxy with vehicle treatment. Box, RUNX2 induction upon Doxy removal from MCF7-tet-off cells (-D). **(D)** To ensure robust sphere formation,CADD522 was added 4 days after cell plating (D4+), and cells were further incubated for 5 days (D5) or for 7 days (D7). **(E)** The cellular invasive ability of MCF7-tet-off cells (left) was evaluated by the 96 Well BME Cell Invasion Assay and xCELLigence systems *in vitro*, respectively. In parallel, similar amount of cells were plated in separate plates and treated with CADD522 for 24 hrs, and Calcein-AM assay was performed (right). Highly metastatic MDA-231 cells were used as a positive control for cellular invasion. Data are presented as fluorescent intensity (Flu). **(F)** The average Cell Index (impedance-based signals) between control and CADD522-treated cells showed little difference in 24 hrs, but decreased by CADD522 in 48 hrs. Medium without cells was used as a negative control.

Three-dimensional sphere cultures of tumor cells from various cancer types including breast [47] and pancreatic [48] cancers are enriched for cancer stem/progenitor cell populations. RUNX2 was recently shown to be upregulated in a subpopulation of MCF7 cells that share molecular characteristics with a more invasive BC phenotype, including genes associated with stem cell renewal and enhanced tumorsphere-forming capacity [13]. We reported that CADD522 treatment resulted in reduction of TGF-β-mediated increase of tumorsphere size in MCF7-tet-off cells that express RUNX2 by removal of Doxycycline (-Doxy) [16]. We now observe that even without TGF-β, MCF7-tet-off (-Doxy) cells (Figure 3A, Supplementary Figure 4E) as well as MDA-231 (Figure 3B, Supplementary Figure 4A & 4E), MDA-468 and MCF7 cells (Figure 3B, Supplementary Figure 4B, 4C & 4E) formed tumorspheres over 7∼10 days. However, CADD522 dramatically decreased the size as well as the number of tumorspheres (Figure 3C). Tumorspheres were severely disrupted a few days after CADD522 treatment at the initial day of cell plating or even when cells were treated with CADD52 at day 4 (D4+) after tight tumorspheres had already formed (Figure 3D). Importantly, treatment with CADD522 did not have a significant influence on mammosphere formation of the MCF10A non-malignant mammary epithelial cells (Supplementary Figure 4D). These results suggest a relatively selective effect of CADD522 on BC cells.

To investigate if CADD522 impairs the invasive phenotype of the BC cells, we performed *in vitro* cell invasion assays in 3D-culture using both 96-well basement membrane extract (BME) cell invasion assay and xCELLigence System (the cell-electrode impedance invasion assay). The MCF7-tet-off (+Doxy) and -off (-Doxy) cells were plated on BME-coated wells and incubated in the presence or absence of CADD522 for 24 hrs. Cellular invasion was promoted by RUNX2 induced by removal of Doxycycline (-Doxy), which was consistent with a previous report [46]. However, CADD522 almost abrogated the invasiveness of both MCF7-tet-off (+Doxy) and MCF7-tet-off (-Doxy) cells (Figure 3E, left). Substantial inhibition of the invasive behavior of MDA-231 cells by CADD522 was also observed in the xCELLigence System that monitors cellular events in real time (Figure 3F). The cell index of the untreated control was 2.3675 ± 0.1214 (mean ± SD), and for the cells treated with CADD522 for 48 hrs 1.6636 ± 0.1845 (*P=0.0456*), respectively. Importantly, CADD522 had little effect on the viability of MCF7-tet-off or MDA-231 cells over 24 hrs (Figure 3E, right), indicating that CADD522 reduces BC cell invasiveness without cellular toxicity.

### CADD522 inhibits RUNX2 transcriptional activity

**Figure 4:**
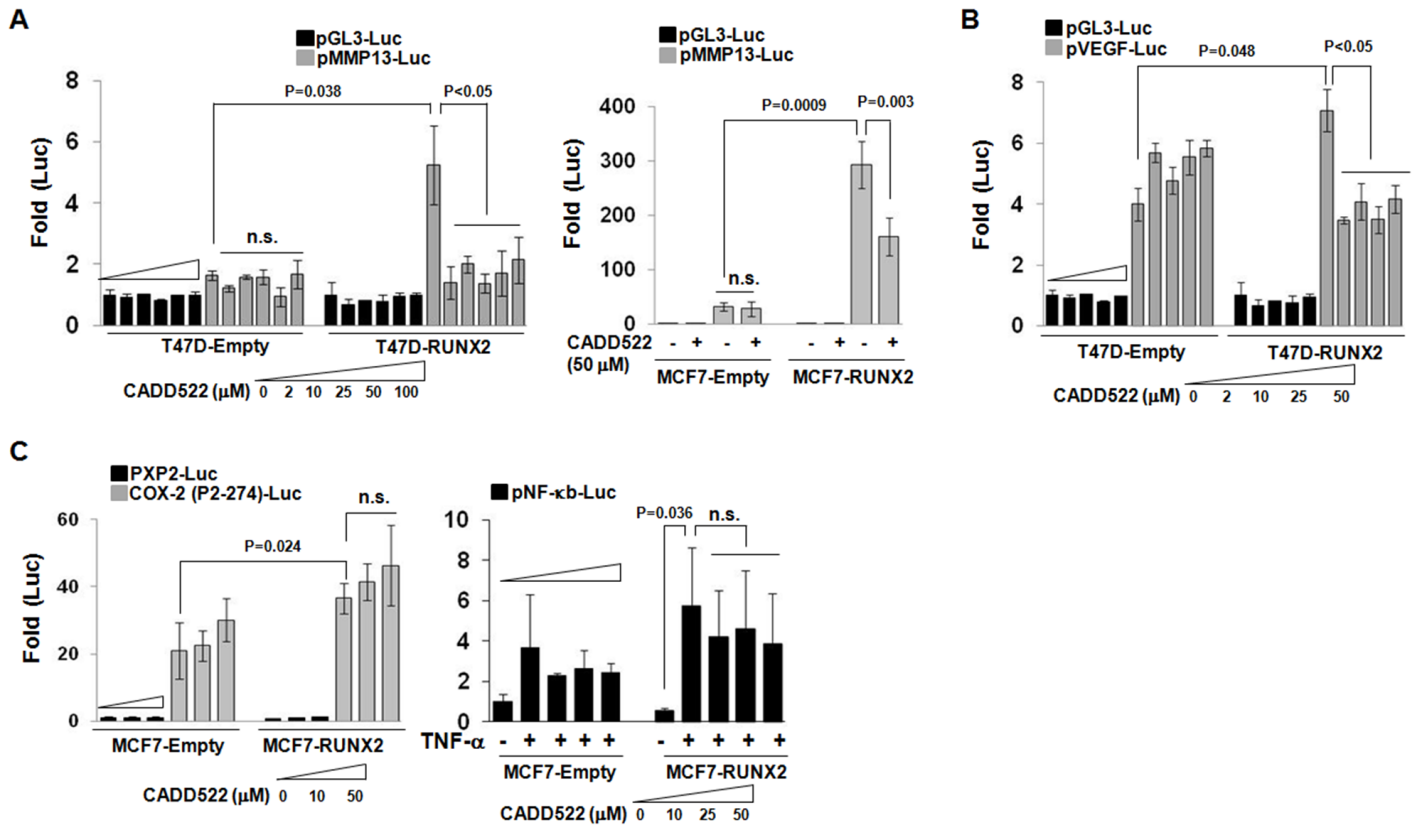
CADD522 decreases RUNX2 transcriptional activity **(A & B)** Ectopic RUNX2-expressing T47D and MCF7 cells with their Empty controls were transfected with indicated luciferase plasmids and treated with CADD522 for 48 hrs (described in Methods). Relative Luc activity (Fold) was calculated from the ratio of target gene Luc activity to pGL3 activity after normalization of pRenilla activity. Increasing concentrations of CADD522 are indicated for each set of data. Data presented as mean ± SD. Experiments were done in triplicate and repeated twice. *P* values are indicated and *<0.05* were considered significant. **(C)** CADD522 does not inhibit the activity of promoters that do not have Runt binding sequences. MCF7-RUNX2 and MCF7-Empty cells were transfected with COX-2 (P2-274)-Luc and control plasmids (PXP2-Luc) (left). In separate experiments, cells were transfected with pNF-kb-Luc plasmids. After 6 hrs, cells were treated with CADD522 (0 ∼ 50 mM) in the absence (1XPBS-0.1% BSA) or in the presence of TNF-a (20 ng/ml) for 24 hrs (right). n.s., not significant. 0.1% DMSO was used as a vehicle control.

CADD522 treatment of ectopic RUNX2-expressing BC cells (T47D-RUNX2 and MCF7-RUNX2) resulted in a dramatic decrease of the promoter-luciferase (Luc) activities of RUNX2 downstream target genes such as MMP13 and VEGF (metastasis markers) (Figure 4A & 4B) and OC (osteogenesis marker) (Supplementary Figure 5A). CADD522 had little effect on the activities of control cells that were stimulated without RUNX2 (T47D-Empty and MCF7-Empty). The inhibition of the promoter-Luc activities of MMP13 in T47D-RUNX2 cells was almost completely blocked by CADD522 at 2 μM, the lowest concentration tested in this study (Figure 4A, left). In particular, the MMP13 promoter-Luc activity of MCF7-RUNX2 cells was about 300-fold higher than pGL3-Luc activity, which was 10-fold higher than MCF7-Empty cells, but CADD522 repressed about 50% of the MMP13 promoter-Luc activity of MCF7-RUNX2 cells (Figure 4A, right). The p6xOSE2-Luc activity was mildly increased in T47D-RUNX2 cells compared to T47D-Empty cells, but the activity was slightly but significantly reduced by CADD522 (Supplementary Figure 5A, left). On the contrary, RUNX2 expression in T47D was associated with about 4-fold lower promoter-Luc activity of the cyclin-dependent kinase inhibitor p21 (Cip1) compared to Empty cells, but CADD522 reactivated the RUNX2-repressed p21 promoter activity (Supplementary Figure 5A, right) [49]. These results indicate that CADD522 negatively regulates transcription of RUNX2 target genes. To determine if CADD522 could inhibit transcriptional activities of other transcription factors that do not belong to the RUNX family, we performed the gene reporter analysis with COX-2 and NF-κb promoter constructs without Runt binding sequences. The activity of the COX-2 P2-274-Luc [50] showed 2-fold increase in MCF7-RUNX2 cells compared to MCF7-Empty cells, which might be through indirect action of RUNX2 (Figure 4C, left), but the activity of pNF-κb-Luc Luc [51] did not show significant increase (Figure 4C, right). Notably, CADD522 had no inhibitory effect on either COX-2 (P2-274)-Luc or pNF-κb-Luc promoter activities, indicating specific inhibition of Runt binding site-mediated transcription by CADD522. In addition, CADD522 did not attenuate the MMP13 promoter activity in BT474 cells that do not express RUNX2 (Supplementary Figure 5B), whereas it decreased significantly the activity in MDA-231 cells that express RUNX2, suggesting the potential specificity of CADD522 for RUNX2.

**Figure 5:**
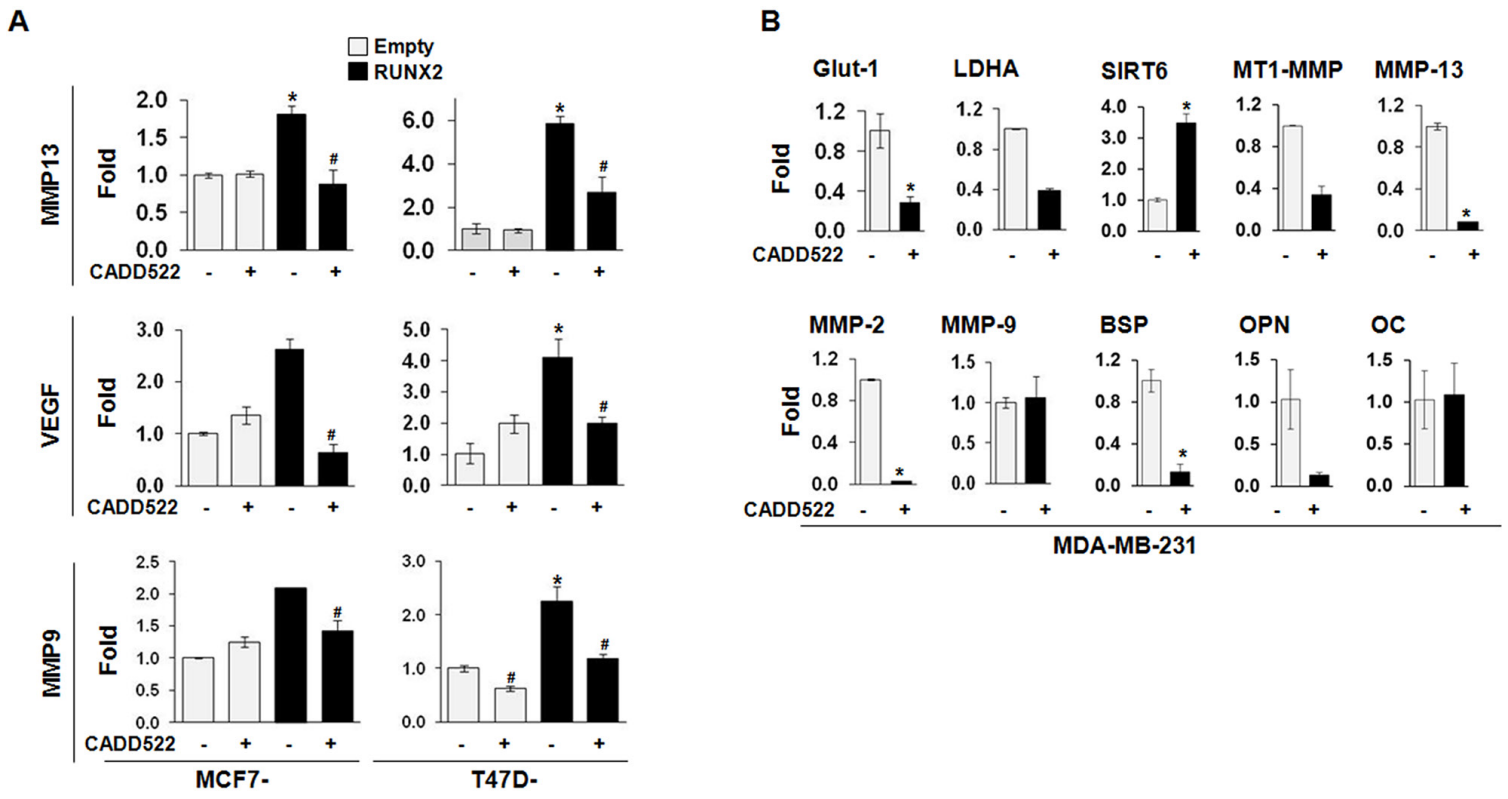
CADD522 inhibits transcription of RUNX2 responsive genes **(A)** Q-RT-PCR analyses of MMP13, VEGF, and MMP9 were performed in ectopic RUNX2-expressing T47D and MCF7 cells. Cells were treated CADD522 (50 μM) for 72 hrs. *, *P<0.05* compared to Empty controls with vehicle alone (-); ^#^, *P<0.05* compared to RUNX2-expressing cells with vehicle control. **(B)** Q-RT-PCR analyses of RUNX2 target genes in MDA-231 cells. Cells were treated with CADD522 (50 μM) for 6 hrs. Similar results were observed in cells treated with CADD522 for 24 hrs (data not shown). *, P<0.05 compared to vehicle control (0.1% DMSO).

Consistent with these observations, CADD522 modulated the mRNA levels of RUNX2 responsive genes such as Glut-1, LDHA, and Sirt6 that regulate glucose metabolism [52], MMP-2, MMP-9, MMP13 and MT1-MMP that regulate tumor invasion/metastasis, and BSP, OPN and OC that regulate osteogenic differentiation. As shown in Figure 5A, CADD522 repressed the mRNA expression levels of MMP13, VEGF, and MMP9 that were upregulated in the ectopic RUNX2-expressing MCF7 and T47D cells. In MDA-231 cells, CADD522 reduced the transcriptional levels of Glut-1 and LDHA but increased the level of Sirt6 (Figure 5B), which was consistent with our previous report that RUNX2 positively regulates Glut-1 and LDHA but negatively regulates Sirt6 expression in glucose metabolism [17]. We observed similar results in MCF7 (Supplementary Figure 6A) and MDA-468 cells (Supplementary Figure 6B). These combined results suggest that CADD522 exerts its inhibitory function on the expression of RUNX2 target genes by inhibiting RUNX2-DNA binding.

**Figure 6:**
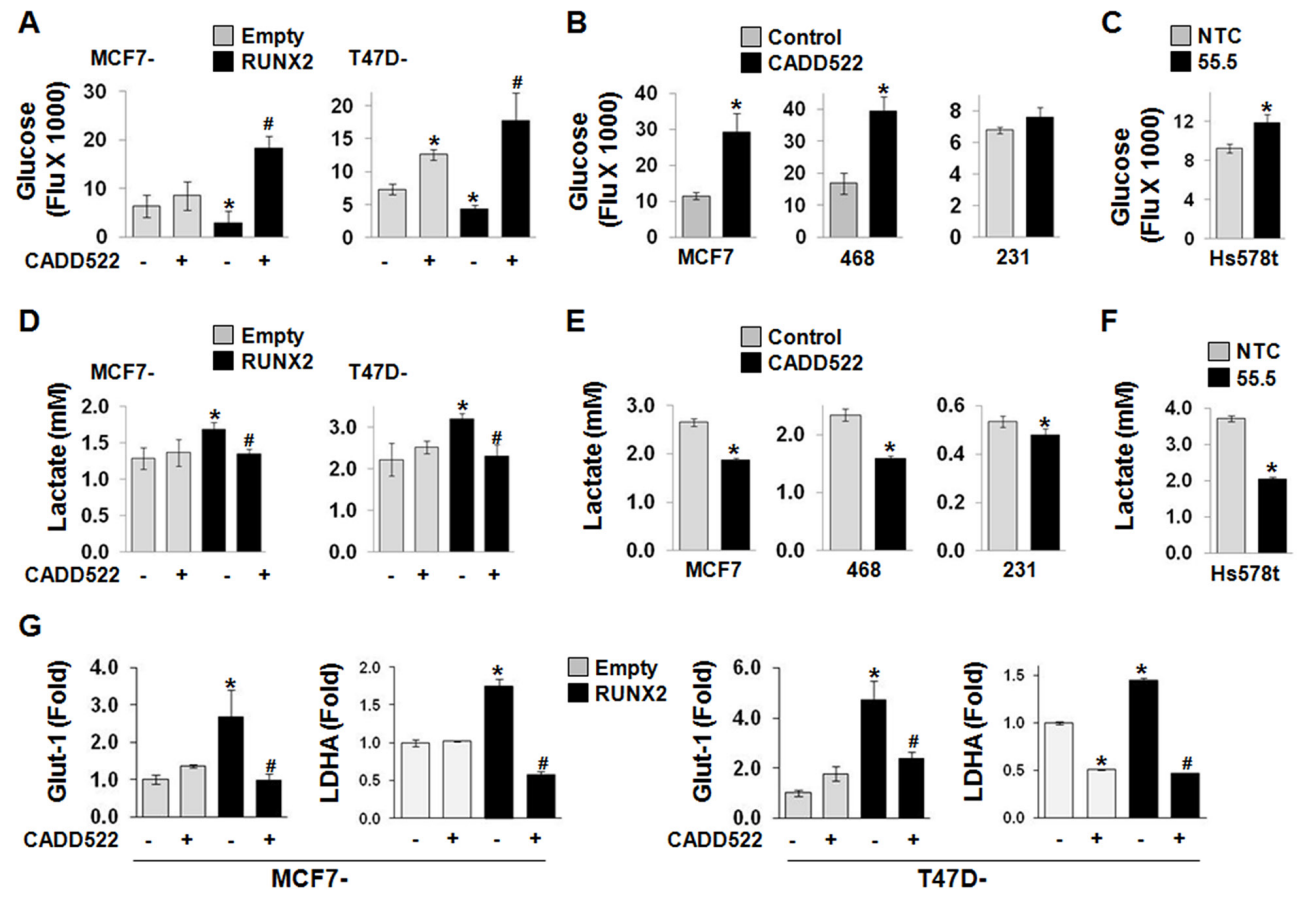
CADD522 alters glucose consumption and lactate production The levels of glucose **(A-C)** and lactate **(D-F)** were measured in the cell culture medium prepared from BC cells treated with or without CADD522 treatment for 6 hrs. (A & D**)** Ectopic RUNX2-expressing MCF7 and T47D cells. (B & E) MDA-231, MDA-468 and MCF7 cells. (C & F) Hs578t cells with RUNX2 KD as described in Materials and Methods. Data presented as mean ± SD. Experiments were done in triplicate and repeated twice. *, *P<0.05* compared to Empty controls with vehicle alone, to untreated control, or to NTC; ^#^, P<0.05 compared to RUNX2 expressing cells with vehicle controls. *P<0.05* considered significant. **(G)** Q-RT-PCR analysis for Glut-1 and LDHA mRNA expressions in ectopic RUNX2-expressing T47D and MCF7 cells. Cells were treated CADD522 (50 μM) for 72 hrs.

In our recent report, RUNX2 promoted glucose metabolism during BC progression by increasing glucose uptake and expression of genes regulating glycolytic pathways such as Glut-1 [17]. To investigate if CADD522 modulates glycolytic phenotypes of BC cells, we examined the levels of glucose and lactate in the cell culture medium. Glucose levels significantly decreased in the medium collected from MCF7-RUNX2 and T47D-RUNX2 cells compared to those in Empty controls (*i.e.,* increased glucose consumption/uptake) (Figure 6A), whereas the level was increased in Hs578t cells with RUNX2 KD (55.5) (*i.e.,* decreased glucose consumption/uptake) (Figure 6C). Similar to RUNX2 KD, the glucose level increased in the medium collected from ectopic RUNX2-expressing MCF7 and T47D cells as well as MCF7 and MDA-468 cells after CADD522 treatment for 6 hrs (Figure 6A & 6B) and 24 hrs (Supplementary Figure 6C). Slight but insignificant inhibition of glucose consumption after CADD522 treatment was also observed in MDA-231 cells (Figure 6B). In contrast, CADD522 significantly reduced the lactate concentration (Figure 6D–6F). Furthermore, we explored the effect of CADD522 on Glut-1 and LDHA transcription by Q-RT-PCR analysis. As reported [17], RUNX2 increased the mRNA level of Glut-1 (up to 5-fold) and LDHA, which was significantly inhibited by CADD522 (Figure 6G). Downregulation of Glut-1 and LDHA mRNA levels by CADD522 in BC cells was shown in Figure 5B, Supplementary Figure 6A & 6B. Diminished Glut-1 protein levels were also observed in MDA-468 and MCF7 cells treated with CADD522 for 72 hrs (data not shown). Therefore, reduction of glucose uptake by CADD522 in BC cells may occur through inhibition of Glut-1 expression to inhibit glycolysis.

### CADD522 upregulates RUNX2 levels through increased RUNX2 stability

**Figure 7:**
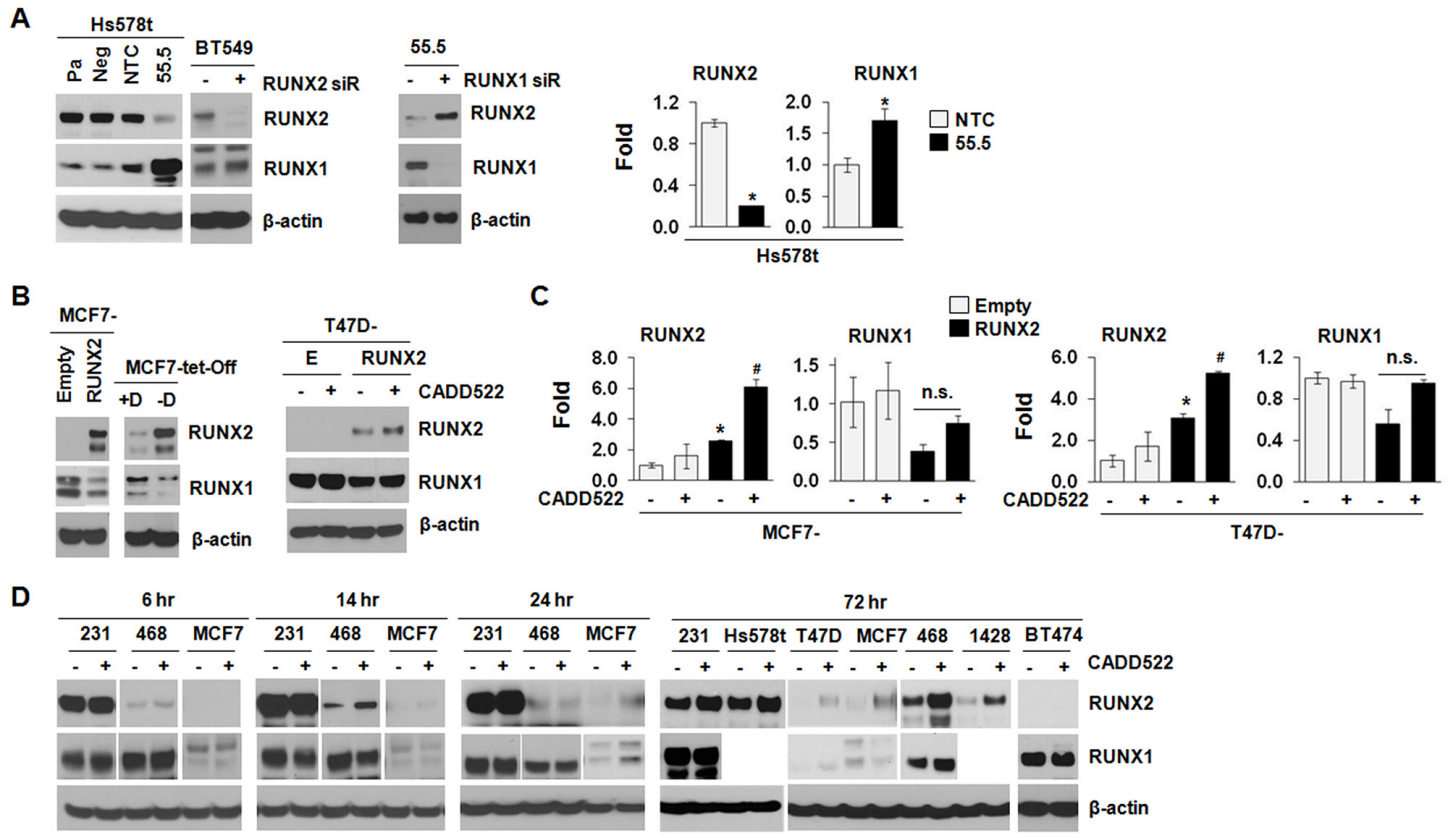
CADD522 modulates RUNX2 expression **(A)**, Compensatory expression of RUNX1 and RUNX2. Left, Pa, parental Hs578t cells; Neg, a negative clone of RUNX2 KD cells (54.5); NTC, a clone for non-targeting control; 55.5, a positive clone for RUNX2 KD. Clones were established under puromycin selection. BT549 cells transfected with RUNX2 or NTC siRNA for 48 hrs. β-actin was used a loading control. Middle, 55.5 cells transfected RUNX1 or NTC siRNA for 48 hrs. Right,Q-RT-PCR analysis in NTC and 55.5 cells to determine the levels of RUNX1 and RUNX2. Data presented as mean ± SD. Experiments were done in triplicate and repeated twice. *, *P<0.05* compared to NTC was considered significant. **(B)** Left, MCF7-tet-off cells were treated with Doxycyclin (+D) for RUNX2 repression and removed (-D) for RUNX2 induction. MCF7-RUNX2 and MCF7-Empty cells were cloned and grown under G418 selection. Right, T47D-Empty (E) and T47D-RUNX2 (RUNX2) cells were treated with CADD522 (50 μM) for 72 hrs, and isolated protein lysates were processed for western blot analysis with indicated antibodies. -, vehicle; +, CADD522. **(C)** Q-RT-PCR analysis in MCF7 and T47D cells expressing ectopic RUNX2 and Empty controls. *, *P<0.05* compared to Empty controls with vehicle alone; ^#^, *P<0.05* compared to RUNX2-expressing cells with vehicle alone.-, Vehicle controls; +, CADD522-treated cells. **(D)** BC cell lines were treated with CADD522 for indicated time periods, and RUNX2 and RUNX1 expression levels were determined by western blot analysis.

RUNX2 and RUNX1 are known to be autoregulated through negative feedback mechanisms targeting their own promoters which stringently regulate expression and function of target genes [53, 54]. mRNA expression levels of RUNX1 and RUNX2 are inversely correlated in skeletal [55] and BC development [56]. In addition, loss of RUNX1 increases RUNX2 mRNA level in acute myeloid leukemia cells [57], and depletion of RUNX2 increases RUNX1 mRNA expression in SaoS-2 and MDA-231 cells [18]. Thus, RUNX1 and RUNX2 might compensate for reciprocal loss during bone formation and BC development in spite of their different structure and specificity [58, 59]. To explore if a similar regulation could be found in BC cells, we examined the RUNX1 levels from the sample sets prepared from the same batches of cDNAs and protein lysates in which we determined RUNX2 levels. As shown in Figure 7A, both 55.5 cells that stably express RUNX2 shRNA and BT549 cells that were transfected with RUNX2 siRNA for RUNX2 knock-down (KD) exhibited increased RUNX1 protein expression compared to the non-targeting control (NTC) (left), which was also observed at the transcriptional level (right). Conversely, RUNX1 KD in the 55.5 cells resulted in RUNX2 re-expression (middle), supporting a potential compensatory mechanism between RUNX1 and RUNX2 in BC. Consistently, the MCF7 and T47D cells that stably express ectopic RUNX2 down-regulated RUNX1 levels compared to the Empty controls (Figure 7B), which was also observed in the RUNX1 mRNA levels (Figure 7C, 2^nd^ and 4^th^ graphs), indicating RUNX1 and RUNX2 could cross-regulate each other in BC cells [56].

Since CADD522 inhibited the transcriptional activation of RUNX2 downstream genes (Figure 4), we speculated that CADD522 could alter RUNX1 and/or RUNX2 levels. We treated T47D-RUNX2 and MCF7-RUNX2 cells with CADD522 (50 μM) for 72 hrs and investigated RUNX2 expression by Western blot analysis. Unexpectedly, however, CADD522 further enhanced both mRNA and protein expression of RUNX2 (Figure 7B & 7C). The increase of RUNX2 proteins by CADD522 was commonly observed in other BC cells (Figure 7D). On the contrary, we did not observe further inhibition of RUNX1 protein and mRNA expression by CADD522 in T47D-RUNX2 (Figure 7B) and MCF7-RUNX2 cells (data not shown). Consistently, RUNX1 protein levels were not altered by CADD522 in most BC cells compared to vehicle controls (Figure 7D), indicating no further cross-regulation by RUNX2 in the presence of CADD522. Therefore, the biological activity of inhibiting RUNX2-DNA binding by CADD522 could be different from RUNX2 KD.

**Figure 8:**
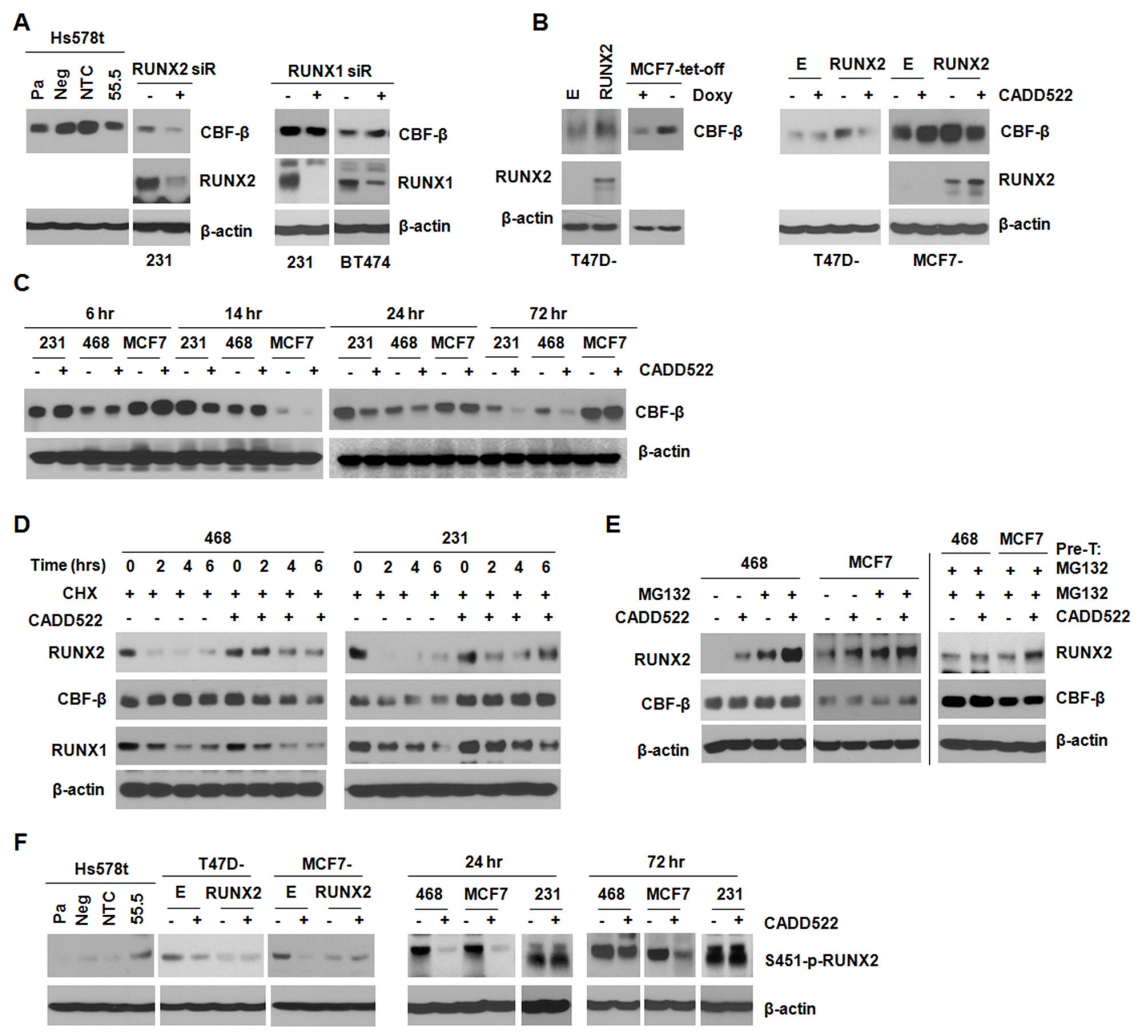
CADD522 increases RUNX2 stability **(A-C)** CBF-β expression was determined by western blot analysis. MDA-231 and BT474 cells were transfected with RUNX1, RUNX2 or NTC siRNA for 48 hrs. CADD522 (50 μM) was treated for 72 hrs. Otherwise, time periods for treatment are indicated in various BC cell lines. Pa, parental Hs578t cells; Neg, a negative clone of RUNX2 KD cells (54.5); NTC, a clone for non-targeting control; 55.5, a positive clone for RUNX2 KD. **(D)** Changes in RUNX2, RUNX1 and CBF-β protein stability under Cycloheximde (CHX, 25 μg/ml) and/or CADD522 (50 μM) treatment for 0, 2, 4 and 6 hrs. **(E)** MDA-468 and MCF7 cells were pre-treated (Pre-T) with MG132 (10 μM) for 1 hr, and then incubated with or without CADD522 in the presence of MG132 for further 18 hrs (left). In separate experiments, MDA-468 and MCF7 cells were pre-treated (Pre-T) with or without CADD522 for 6 hrs, and MG132 was added for further incubation for 18 hrs (right). **(F)** S451-p-RUNX2 levels were determined in MCF7 and T47D cells expressing ectopic RUNX2 after CADD522 (50 μM) treatment for 72 hrs. MDA-231, MDA-468 and MCF7 cells were treated with CADD522 for 24 hrs or 72 hrs.

Studies have suggested that the non-DNA-binding subunit of mammalian core binding factor CBF-β stabilizes the RUNX proteins in a conformation that is favorable for DNA binding [60, 61], which facilitates RUNX-mediated gene transcription. Therefore, CADD522 might regulate CBF-β expression to inhibit transcriptional activity of RUNX2. The protein level of CBF-β decreased in Hs578t (55.5) and MDA-231 cells with RUNX2 KD compared to the non-targeting controls, whereas the level did not change in MDA-231 and BT474 cells with RUNX1 KD (Figure 8A). In addition, ectopic RUNX2 in both T47D and MCF7 cells resulted in increased CBF-β expression, but CADD522 reduced the CBF-β level (Figure 8B). In MDA-231, MDA-468 and MCF7 cells, CBF-β levels were increased by CADD522 after 6 hrs of treatment, but the levels decreased after 24 hrs (Figure 8C). These results suggest that RUNX2, but not RUNX1 might regulate CBF-β expression, which might be modulated by CADD522 in a BC cell type- and time-dependent manner.

RUNX2 stability is regulated by post-translational events such as ubiquitination, phosphorylation and acetylation [62, 63]. When RUNX2 is not bound to DNA, it is either targeted to sub-nuclear compartments [63] or rapidly degraded [64]. To explore if CADD522 could alter RUNX2 stability, we treated MDA-468 and MDA-231 cells with Cycloheximide (CHX), a protein synthesis inhibitor, in the presence or absence of CADD522 (50 μM). We found that CADD522 increased RUNX2 stability in both MDA-468 and MDA-231 cells by delaying protein degradation (Figure 8D). In contrast, the stability of RUNX1 or of CBF-β was not increased by CADD522 in the presence of CHX (Figure 8D). Moreover, RUNX2 is normally degraded by the ubiquitin-proteasome pathway. Therefore, we co-treated MDA-468 and MCF7 cells with CADD522 and the proteasome inhibitor MG132 (10 μM) for 18 hrs. RUNX2 protein stability was increased by MG132 alone, which was consistent with previous findings [65], and further increased in the presence of CADD522 (Figure 8E, left). The increase appeared additive for both MDA-468 and MCF7 cells. In MDA-468 and MCF7 cells pre-treated with MG132 for 1 hr and then co-treated with or without CADD522 for 18 hrs, the RUNX2 level was clearly increased in MCF7 cells by CADD522, and slightly increased in MDA-468 cells (Figure 8E, right), whereas CBF-β protein stability was not altered by MG132. In addition, ubiquitin expression was decreased by CADD522 in MDA-468 cells and MCF7 cells (data not shown). These results indicate that both proteasome-dependent and -independent pathways might be involved in the increase of RUNX2 stability by CADD522.

Phosphorylation is an important post-translational mechanism for regulation of RUNX2 stability and activity. It has been shown that the alanine substitution at the S451 residue (S451A) of RUNX2 reduces phosphorylation, leading to decreased DNA binding activity [66]. cdc2 (CDK1) is known to phosphorylate RUNX2 on Ser-451 *in vitro*, but little is known about the functional significance and regulation of the S451 phosphorylation of RUNX2 in BC. RUNX2 KD cells (55.5) expressed higher level of S451 phosphorylation compared to the non-targeting controls (Figure 8F), but T47D-RUNX2 and MCF7-RUNX2 cells expressed lower levels of the S451-p-RUNX2 compared to the Empty controls, indicating that RUNX2 could regulate its stability and activity through regulation of phosphorylation at the S451 residue. In contrast, in Empty controls, MDA-468 and MCF7 that express relatively low levels of RUNX2, CADD522 clearly reduced the S451-p-RUNX2 levels, which might contribute to the increased stability of RUNX2 protein. However, little difference was observed in cells that express high levels of RUNX2 (T47D-RUNX2, MCF7-RUNX2 and MDA-231), implying that a negative feedback mechanism suppressing further S451 phosphorylation might be activated to balance the higher levels of RUNX2 by CADD522 or that BC cells might have different biological response to CADD522, which might be related to cellular levels of RUNX2. Furthermore, dynamic shuttling of RUNX2 between cytoplasmic and nuclear compartments could be linked to its gene regulatory activity, promoting post-translational modifications of RUNX2 and/or interactions with co-factors. It is known that RUNX2 distribution and compartmentalization between the cytoplasm and nucleus could be altered by Paclitaxel treatment [67]. In our western blot analysis, however, we did not observe that CADD522 blocked or attenuated nuclear translocation of RUNX2 protein (Supplementary Figure 7C). Taken together, in addition to its interference with RUNX2-DNA binding, CADD522 might increase RUNX2 stability and reduce phosphorylation and CBF-β levels, which might be through direct or indirect mechanisms.

### CADD522 suppresses *in vivo* tumor growth and metastasis

**Figure 9:**
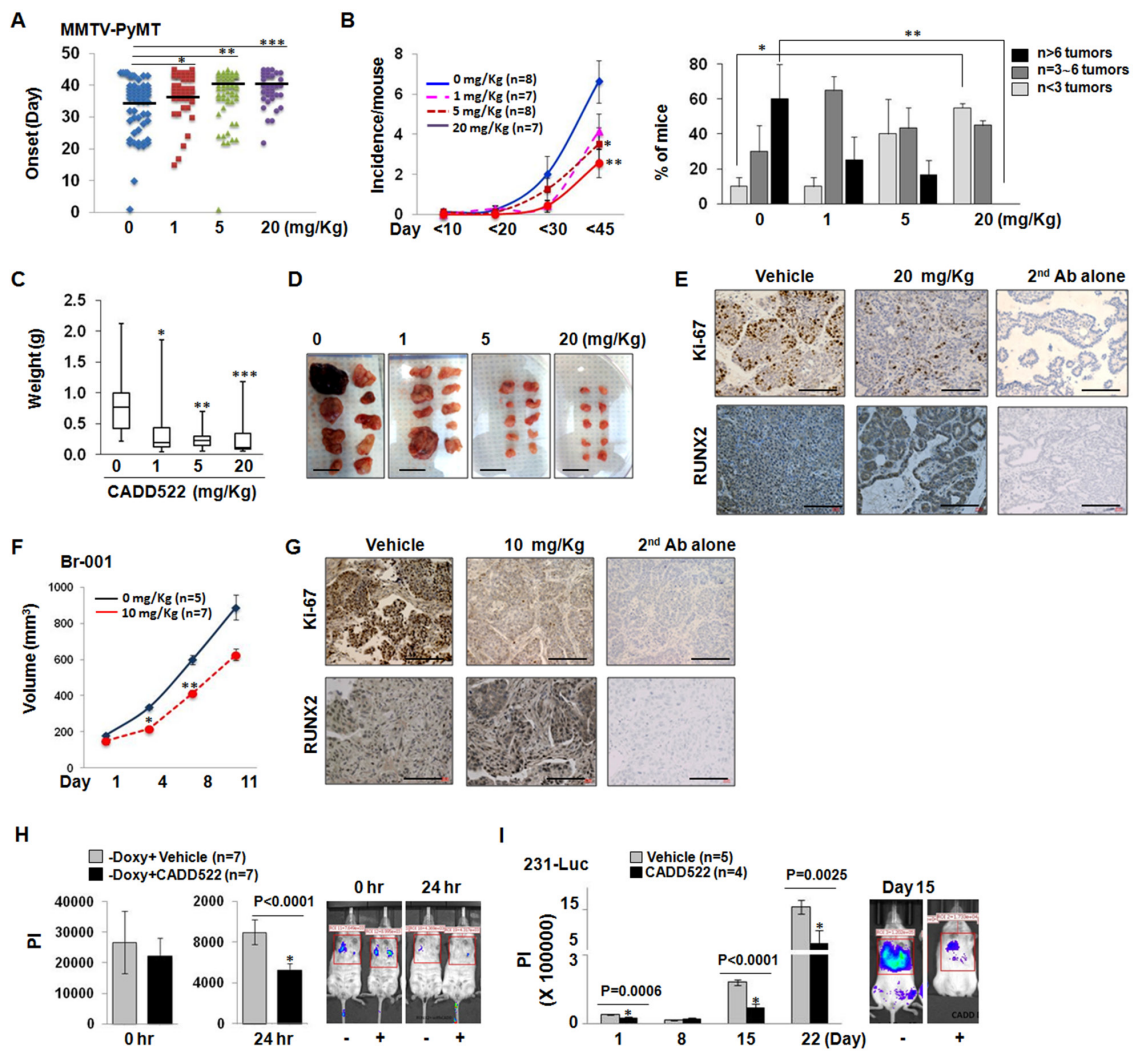
CADD522 suppresses *in vivo* BC cell growth and metastasis **(A)** MMTV-PyMT female mice were injected with CADD522 for 45 days, and detection of first palpable tumors (tumor onset) was depicted in Scatter plot. Vehicle (10% DMSO in 90% PBS) was injected in mice for 0 mg/kg CADD522. Data represent combined results from two separate experiments. The range of vehicle control was 1 ∼ 44, of 1 mg/kg CADD522 was 15 ∼ 45, of 5 mg/kg CADD522 was 1 ∼ 45, and of 20 mg/kg CADD522 was 22 ∼ 45. Mode/Frequency of vehicle control was 39/9, of 1 mg/kg CADD522 was 43/8, of 5 mg/kg CADD522 was 43/11, and of 20 mg/kg CADD522 was 40/9. *, *P=0.0086*; **, *P=0.0051*, ***, *P=0.0007* (Mann-Whitney test). *P<0.05* considered significant. Black lines, median values in each group. **(B)**, Left, Tumor incidence (tumor number per mouse) determined by palpation of all 10 mammary glands from day 1 to day 90 (described in Materials and Methods). Data presented as mean ± SE. *, *P=0.037*; **, *P=0.008* (Student’s *t-*test). Right, Fraction of mice with tumors (%) at the final day. Note, no mice treated with 20 mg/Kg of CADD522 had over 6 tumors. *, *P=0.007*; **, *P=0.05* (Student’s *t-*test). **(C)**, Median tumor weight with min/max values depicted via Box/Whisker plot. Tumors were excised from control mice (n=13), 1 mg/Kg (n=15), 5 mg/Kg (n=20) and 20 mg/Kg CADD522-treated mice (n=17), and weighed after mice were sacrificed. *, *P=0.0034*; **, *P=0.0002*, ***, *P=0.0005 (Mann-Whitney test*). **(D)**, Representative tumors excised from a mouse are shown. Scale bar, 2 cm. **(E)**, Analysis of Ki67- and RUNX2-positive proliferating cells within MMTV-PyMT mice. Shown are representative fields from 3 separate tumors per group. 2^nd^ antibody alone was used as a negative control. Scale bar = 100 mm. **(F)**, TNBC-PDX Br-001-bearing mice were treated with CADD522, and tumor volume (mean ± SEM) was determined for 11 days. *, *P=0.002*; **, *P=0.005* (Student’s *t-*test). **(G)**, IHC analysis of Br-001 tumors with Ki-67 and RUNX2 antibody. Scale bar = 100 mm. The lung retention of MCF7-tet-off-Luc (-Doxy) cells that express ectopic RUNX2 **(H)** and 231-Luc cells **(I)** was monitored by BLI analysis. Vehicle or 10 mg/kg CADD522 was administrated into NSG mice. Data presented as mean ± SE of PI (Photon Intensity) (Student’s *t-*test), and *P<0.05* compared with control were considered significant (left). Left, Representative images.

To assess the *in vivo* activity of CADD522, we used the MMTV-PyMT transgenic model, in which the polyoma middle-T oncogene is activated under control of the mouse mammary tumor virus promoter (MMTV-PyMT) [68]. This model was suitable for our purpose since it mimics human breast cancer from the stages of initial hyperplasia to ductal carcinoma in situ and invasive ductal carcinoma [69]. Conditional RUNX2 deletion in the MMTV-PyMT transgenic mouse model of BC has been shown to delay tumor incidence and enhance overall survival [70]. We observed that MMTV-PyMT females developed palpable mammary gland tumors at approximately 5 ∼ 6 weeks of age, and high levels of RUNX2 were expressed in tumor tissues compared to normal mammary gland in age-matched control mice over 10 weeks of observation (Supplementary Figure 8A). RUNX2 expression also increased with disease progression. We randomly assigned 45-day-old mice to control and CADD522 treatment groups. Tumor incidence was measured every 1 to 2 days, and mice were observed for up to 90 days of age (total of 45 days of CADD522 treatment) at which time the mice were sacrificed, tumor volume was calculated as described in the Materials and Methods, and tumors were excised and weighed. The intraperitoneal (*i.p.*) administration of CADD522 into the MMTV-PyMT mice (up to 20 mg/kg) delayed tumor development and reduced tumor burden in transgenic MMTV-PyMT mice. We observed CADD522 significantly delayed the onset of the tumors (Figure 9A). Statistical analysis by the two-sample Wilcoxon rank-sum (Mann-Whitney) test revealed significance between vehicle control group and each of the CADD522-treated groups (*p<0.05*).

CADD522-treated MMTV-PyMT mice exhibited significant decrease in tumor incidence compared to vehicle control animals. The incidence per mouse at the final day was 6.63 ± 1.05 (mean ± SEM) in vehicle-treated mice, 4.14 ± 0.88 in 1 mg/Kg, 3.05 ± 2.57 in 5 mg/Kg (*P=0.037*), and 2.57 ± 0.72 in 20 mg/Kg CADD522-treated group (*P=0.008*) (Figure 9B, left). The fraction (%) of mice with over 6 tumors was 60 ± 20% (mean ± SEM) in control group, but no mice in 20 mg/Kg CADD522-treated group had over 6 tumors (*P=0.05*) (Figure 9B, right). Mice with less than 3 tumors were only 10% of the control group, but increased to 40% and 55% of 5 mg/Kg and 20 mg/Kg CADD522-treated groups, respectively. We also found a reduction in the tumor weight (Figure 9C) in CADD522-treated mice. Representative tumors are shown in Figure 9D. Since we observed that CADD522 had an inhibitory effect on *in vitro* BC cell growth, we explored if CADD522-driven inhibition of mammary tumorigenesis would be due to an effect of CADD522 on cell proliferation. IHC analysis of Ki67 (proliferative index) in tumor sections showed that staining of Ki67 was much weaker in tumors from CADD522-treated mice than in control mice (Figure 9E, upper panel).

Patient-derived tumor xenografts (PDX) are powerful pre-clinical models to recapitulate the diversity of human tumors [71]. In a TNBC-PDX Br-001 model, RUNX2 expression was positive during continuous passage from P1 to P3 (Supplementary Figure 8C), and a significant decrease of tumor volume was observed in the CADD522-injected Br-001-bearing athymic mice (10 mg/kg, *i.p*.) (Figure 9F). In this PDX model, Ki-67 expression was markedly reduced in mice injected with CADD522 (Figure 9G). In both MMTV-PyMT mammary and PDX tumors, higher RUNX2 staining was observed in most of the tumor sections from CADD522-injected mice compared to vehicle control mice (Figure 9E & 9G, lower panel), but only negligible caspase-3 staining and no necrosis were observed in the tumor sections from vehicle control or CADD522-injected mice (data not shown), which suggest that CADD522 might delay detectable tumor growth in mice without inducing apoptosis.

Bioluminescence Imaging (BLI) analysis is useful for sensitive *in vivo* tumor detection and quantification, and permits earlier detection of tumor growth and metastasis. To further examine the effects of CADD522 on the metastasis of breast cancer, we performed BLI analysis using BC cell lines stably expressing firefly luciferase (MCF7-tet-off-Luc (-Doxy) and 231-Luc) after intravenous delivery of cells into NSG mice. MCF7-tet-off-Luc (-Doxy) cells express RUNX2 when the cells are maintained without Doxycycline (Doxy) [16, 17]. CADD522 had no influence on metastatic homing of the MCF7-tet-off-Luc (-Doxy) cells to the mouse lungs (Figure 9H, left) but significantly reduced the lung retention of the cells (Figure 9H, right). As the MCF7-tet-off-Luc (-Doxy) cells did not form stable tumors in the lungs of NSG mice (data not shown), we injected 231-Luc cells in the tail vein of mice and repeated the BLI analysis. Our results show that colonization and outgrowth of the 231-Luc cells were significantly lower in the CADD522-treated groups than in the vehicle-treated groups (Figure 9I), indicating that CADD522 inhibits experimental metastasis of BC cells *in vivo*. Doses of CADD522 up to 20 mg/kg in MMTV-PyMT mice (Supplementary Figure 8B), and 10 mg/kg of CADD522 in the nude (Supplementary Figure 8D) and NSG mice (Supplementary Figure 8E) did not significantly decrease body weight or influence the general health of animals.

## DISCUSSION

### Therapeutic potential of CADD522 for BC *in vitro* and *in vivo*

The context-specific nature of many transcription factor dependencies can lead to the identification of a large number of therapeutic opportunities [72]. There are many possible therapeutic targets in transcription factor signaling but demonstrating therapeutic efficacy has been a barrier to progress [35]. In this study, we have tested CADD522 as a small molecule compound that inhibits RUNX2-DNA binding, transcriptional activity, proliferation and tumorsphere formation of BC cells. The CADD522compound also delayed and/or inhibited tumor formation in an aggressive spontaneous model of mammary carcinoma and reduced tumor growth of a RUNX2-expressing patient-derived xenograft (PDX), supporting that CADD522 has therapeutic potential for BC *in vitro* and *in vivo*.

The mechanism of tumor formation in MMTV-PyMT transgenic mice has been well characterized and reported to involve the activation of PI3K/Akt [73, 74], Src [75] and Ras [76, 77] signaling pathways. Matrix metalloprotease family members such as MMP2, MMP3, and MMP13 are also known to be increased in PyMT tumors [76, 78]. Interestingly, a large cassette of these genes are directly or indirectly associated with transcriptional regulators including RUNX2 [17, 79, 80]. Thus, it would not be surprising that tumor growth was delayed in CADD522-treated MMTV-PyMT mice. It remains to be determined which previously identified genes that function in tumor formation in MMTV-PyMT mice might be influenced (or regulated) by CADD522. PyMT tumors initiate as Luminal B type (ER-positive/PR-positive/HER2-positive) tumors but become ER-negative with time [69]. ER is expressed by stroma at early stage, and tumors at late stage show very dense carcinoma cells with few stromal elements. Analysis of H&E staining of tumor sections revealed that CADD522 treatment in the MMTV-PyMT mice maintained tumors at a well-differentiated stage of the MMTV-PyMT mammary tumor, whereas vehicle treated tumors consisted mainly of a solid sheet of tumor cells (Supplementary Figure 8F), indicating that CADD522 might prevent BC progression in the PyMT model of BC. In contrast, little difference in morphology between control and CADD522-treated PDX tumors was observed even though CADD did reduce Ki67 staining. The difference could be because MMTV-PyMT tumors initiate as glandular, ER-positive and progress to ER-negative tumors, but the PDX tumor was a TNBC, which was already poorly differentiated before treatment. These results indicate that CADD522 could prevent early tumor development and also inhibit growth of already established tumor cells.

RUNX2 is an essential factor in BC growth and metastasis [16, 66, 81-86], but it was reported that no RUNX2 was expressed in cell lines derived from ‘luminal-like’ tumors [30]. Our western blot analyses show that RUNX2 was clearly observed in luminal-type MCF7 and T47D cells [16]. Therefore, in most BC cell lines, the anti-cancer activity of CADD522 could be, in part, through its targeting of RUNX2-DNA binding. However, CADD522 may impact BC in multiple ways, including off-target effects. The results of our western blot analysis revealed that HER2-positive SKBr3 cells express no RUNX family proteins. However, CADD522 treatment of SKBr3 cells for three weeks reduced cell survival less than 50%, indicating potential off-target effects of CADD522. Substantial suppression of cell survival (< 5 %) in the presence of CADD522 was also observed in HER2-posive BT474 cells that express only RUNX1. We observed that the protein expression of HER2 and CBF-β in BT474 was diminished by CADD522 treatment (Supplementary Figure 7A), indicating that the therapeutic potential of CADD522 could be through both on-target (interference of Runt-DNA binding) and off-target effects (disruption of HER2 signaling pathway). In addition, recent findings of McDonald et al. that no RUNX3 was detected in mammary epithelial populations sorted by FACS with CD29 and CD24 surface markers [41] indicate that potential toxicity driven by CADD522 could be minimal in BC patients. However, the potential inhibition of RUNX3 by CADD522 could be toxic to normal tissue of other organs in cancer patients. Our results showed that CADD522 (0 ∼ 100 μM for 72 hrs) did not cause cytotoxicity or significant cell growth inhibition of non-tumorigenic cell lines. In addition, CADD522 has shown no apparent side effects to the mice in our studies. These results suggest that CADD522 might not be toxic to normal cell growth. Furthermore, our recent report that CADD522 activates theHippo tumor suppressor pathway in luminal breast cancer [16] suggests a compensatory pathway to overcome the potential drawback of RUNX3 inhibition by CADD522.

### Specificity of CADD522 for RUNX2

It would be most desirable to develop therapeutic agents targeting transcription at the single gene level. However, it is also arguable whether there is a real need to develop single-gene targeted agents considering that the growth and survival of cancer cells are often driven by aberrant expression of a group of related genes that are often co-regulated by common transcription factors such as RUNX2 or p53 [40]. A previous report has shown that all three RUNX proteins commonly bind to OSE2 of human OC promoter that include Runt binding sites [87]. In the report, DNA binding activity determined by the electromobility shift assay was found to be similar among all RUNX proteins, but RUNX2 exhibited the highest promoter activity for p6xOSE2-luc [87]. We determined DNA-binding activity of RUNX proteins by D-ELISA using nuclear extracts isolated from HBME-1 human bone marrow-derived endothelial cells, the oligonucleotides containing sequences for the OSE2, and specific antibodies to capture each DNA-bound RUNX protein as reported [43, 44]. We observed that CADD522 exhibited the most prominent inhibition in RUNX2-DNA binding among three RUNX proteins. Although unequal expression levels of each RUNX protein and different antibody specificity could prohibit accurate comparison of the DNA binding activity among RUNX proteins, these results suggest that CADD522 might be relatively specific for RUNX2. Although it is not clear how CADD522 is more specific for RUNX2 inhibition, it could be speculated that different three-dimensional architecture and dynamics in response to RUNX protein-cofactor binding or altered efficacy in RUNX1- or RUNX3-DNA binding relative to RUNX2 might influence CADD522 fit into the DNA-Runt binding pocket, which would yield a distinctive DNA binding affinity among RUNX proteins [23], and may have directly contributed to delay of tumor growth. In addition, RUNX proteins control the expression of different target genes to achieve tissue-specific gene expression and phenotype (*e.g.*, MMP13 for RUNX2, Platelet factor 4 for RUNX1 and Bcl-2 for RUNX3) [88-90]. Therefore, future studies will reveal the relative specificity of CADD522 for RUNX proteins using D-ELISA with DNA oligonucleotides including the Runt binding site of RUNX1 or RUNX3 downstream targets and by gene-specific promoter-reporter assays for RUNX1 and RUNX3 targets. D-ELISA and promoter-reporter assays for other transcription factors (*e.g.,* p53 or HIF-1ɑ) and their downstream targets will help further define CADD522 specificity for RUNX2.

In an effort to address the relative selectivity of the CADD522 in RUNX proteins, we examined mRNA expression levels of RUNX1 or RUNX2 target genes in MCF7 cells overexpressing RUNX1 (MCF7-RUNX1) or RUNX2 (MCF7-RUNX2) by Q-RT-PCR analysis. MMP13, VEGF, MMP9 and Glut-1 are known to be downstream targets of RUNX2 [17, 42, 91, 92], whereas transcriptional regulation of these genes by RUNX1 has not been well documented (0∼3 publications for RUNX1 and MMP13, MMP9, or Glut-1, and 20 publications for RUNX1 and VEGF in the NCBI-PubMed search results). VEGF is reported to be repressed by RUNX1 in acute myeloid leukemia [93], whereas PF4 (platelet factor 4) is upregulated by RUNX1 in human erythroleukemia cells [88]. MMP9 and VEGF are known to be downstream targets of RUNX2 [45, 46] but little has been reported on relevance of RUNX2 and PF4 or of RUNX1 and MMP9. All these genes have the runt-domain binding element in their promoters. Transcriptional levels of VEGF, Glut-1, and MMP13 increased in both MCF7-RUNX1 and -RUNX2 cells, which were also inhibited by CADD522 treatment (Supplementary Figure 9). PF4 mRNA levels increased in MCF7-RUNX1, but not in MCF7-RUNX2 cells, and MMP9 levels increased in MCF7-RUNX2 but not in MCF7-RUNX1 cells. Interestingly, CADD522 suppressed the MMP9 level that was upregulated by RUNX2, but not the PF4 level that was increased by RUNX1. In addition, CADD522 further enhanced the RUNX2 level in MCF7-RUNX2 cells, whereas CADD522 partially but significantly inhibited the RUNX1 level in MCF7-RUNX1 cells. These results support the relative CADD522 selectivity for RUNX2.

Even though inhibition of RUNX1-DNA binding by CADD522 was weaker than RUNX2-DNA binding in D-ELISA, we do not exclude the possibility that the *in vitro* and *in vivo* efficacy of CADD522 in BC models could also involve inhibition of RUNX1-DNA binding. From our results, RUNX1 was ubiquitously expressed in all of the BC cell lines with the exception of SKBr3. Of note, Browne et al. recently reported an association of RUNX1 with breast cancer progression in MMTV-PyMT transgenic mice [24], and some studies have shown that ablation of RUNX1 in other epithelial cancers dramatically reduces tumor burden [94]. In addition, small-molecule inhibitors of the RUNX1-CBFβ interaction reduce tumor burden in murine leukemia models [95, 96]. Therefore, even if CADD522 is not strictly specific for RUNX2-DNA binding, the potential of CADD522 for BC therapeutics is still significant; CADD522 could be a strong anticancer drug due to its potential inhibition of both RUNX1 and RUNX2-DNA binding.

RUNX1 and RUNX2 exhibit different cellular functions as a result of multivalent interactions with diverse protein partners, but high similarities in the DNA binding motifs and their DNA binding elements [97-99] suggest that they might have functional similarities in BC. Due to the cross-regulatory mechanism between RUNX1 and RUNX2, specific suppression of RUNX2 might thus induce a reciprocal increase in RUNX1 in BC. Our results show that CADD522 inhibited the transcriptional activation of RUNX2 downstream genes by interfering with RUNX2-DNA binding. As a consequence, CADD522 treatment could result in an increase of RUNX1 and/or RUNX2 to compensate for the functional loss of RUNX2, which is normally necessary to mediate BC growth, survival or metastasis. We observed an increase of RUNX2 by CADD522 in most BC cells, which was regulated via translational and transcriptional regulatory mechanisms, whereas CADD522 had little influence on RUNX1 expression, indicating that CADD522 could overcome a potential drawback derived from specific RUNX2 knock-down or knock-out. The DNA-unbound RUNX2 might recruit other co-factors to its own promoter or interact with other proteins to activate RUNX2 transcription and translation to reduce cellular need for RUNX2, which is functionally inactive in the presence of CADD522. All these results highlight the potential of CADD522 in future BC therapeutics. More in-depth exploration is warranted to fully elucidate the molecular mechanisms of CADD522 function in BC.

### CADD522 inhibition of RUNX2-DNA binding: mechanistic consequences

A potential mechanism by which CADD522 could exert an inhibitory effect on RUNX2-DNA binding might be through its physical interference with the RUNX2 and DNA binding pocket. As proposed by Pregizer et al., RUNX2-mediated transcriptional control is restrained by confining the access of RUNX2 to its genomic targets [64]. The inverse expression of RUNX2 and OC during osteoblast differentiation may be due to the minimal occupancy of RUNX2 at the genomic locus of the OC promoter even when RUNX2 protein amount was at maximal level. Similarly, the occupancy of RUNX2 at the genomic loci of its target gene promoters might be prevented by CADD522, leading to loss of function as a transcription factor. Nevertheless, it is not clear whether CADD522 directly interacts with RUNX2. The growth of T47D and MCF7 cells was delayed under CADD522 treatment but the growth rate was similar to that of control cells in fresh growth medium without CADD522 (data not shown). These results indicate that CADD522 might not irreversibly bind to DNA and/or RUNX2. CADD522 could interfere with RUNX2 binding to DNA through simple insertion into the RUNX2-DNA binding pocket without physical or chemical interactions. Whether or not CADD522 interacts with and inhibits RUNX2-DNA binding will be investigated with recombinant RUNX2 protein in a future study.

CADD522 might play an inhibitory role for RUNX2-DNA binding through inhibition of glucose uptake (consumption) leading to cell cycle arrest at the G1 or G2/M phase. It has been shown that glucose stimulated RUNX2 phosphorylation and activated DNA binding in endothelial cells by promoting cell cycle progression through both G2/M and G1 phases with entry into S-phase. Starvation conditions (the absence of nutrients and growth factors) delayed the cell cycle at the G1 phase or G2/M phase, which resulted in no DNA binding [66, 82]. Our results show that BC cells treated with CADD522 exerted significant inhibition in glucose consumption, indicating that cells treated with CADD522 might be exposed to glucose deprived conditions that could delay cell cycle progression and reduce RUNX2-DNA binding. Moreover, RUNX2 levels might be regulated in multiple phases of the cell cycle, which is also in a cell type-dependent manner. It has been shown that RUNX2 was reduced at the G1 phase in endothelial cells [66, 82], but RUNX2 levels were found to be up-regulated with quiescence (G0/G1) in MC3T3 osteoblastic cells [100]. Our results show that RUNX2 levels were increased, and the cell cycle was arrested at G1 or G2/M by CADD522 in BC cells. Therefore, targeting RUNX2-DNA binding by CADD522 in BC cells might disrupt the coordinately regulated RUNX2 levels with the cell cycle machinery by modulating RUNX2 mRNA transcription and protein stability.

The inhibition of RUNX2-DNA binding by CADD522 could also be mediated through down-regulation of CBF-β. Core binding factor (CBF) is a heterodimer composed of one of three DNA-binding RUNX proteins and CBF-β, the non-DNA-binding partner. Recent studies showed that CBF-β was required for the malignant phenotype of prostate and ovarian cancers [61, 101]. CBF-β is ubiquitously expressed across different tissue types [102], but the genes encoding CBF-β are amplified in human granulocytic sarcoma [103], suggesting that CBF-β can be oncogenic. Accordingly, CBF-β positively regulates genes involved in the malignant phenotype, including VEGF and MMP9 [46, 84]. However, CBF-β is known to have dual roles as an activator and a repressor of transcription in a cell type-specific and promoter-dependent manner [104].

RUNX2 protein phosphorylation directly correlates with its protein stabilization. Phosphorylation of S451 (S472 in the murine RUNX2) resides within the C-terminal transcription inhibition domain of RUNX2 and hence it was suggested that its phosphorylation might inhibit RUNX2 activity by ubiquitination and proteasomal degradation of RUNX2 [105]. A report from Shen et al. [106] that mutation of serine-472 to alanine decreased the phosphorylation of RUNX2 protein, leading to increased stability and transcriptional activity in osteogenic progenitor C3H10T1/2 cells supports the presumption. However, Qiao et al. [66] reported contradictory results that in HBME-1, the alanine substitution at the S451 residue of RUNX2 exhibited reduced phosphorylation as well as DNA binding activity relative to wild type RUNX2. In addition, the RUNX2 Ser451 mutant was less potent than wild type RUNX2 at stimulating anchorage-independent growth of NIH3T3 fibroblast cells. From our results, CADD522 reduced the S451 phophorylation in BC cells with low RUNX2 expression but did not change the level in BC cells with high RUNX2 expression. Therefore, the outcomes of S451 phosphorylation might be cell type-dependent.

In summary, we report here that CADD522 plays a suppressive role in BC cell growth and survival in various BC cell lines and animal models of tumor growth and metastasis. The suppressive effects of CADD522 on the initiation and progression of BC might be primarily due to its inhibitory effect on RUNX2-DNA binding, leading to significant reduction in RUNX2-mediated target gene transcription. CADD522 upregulates RUNX2 expression, whereas it downregulates levels of CBF-β and S451-RUNX2 phosphorylation, which might contribute to increased RUNX2 stability. Results presented in this study therefore suggest that CADD522 could be a potential therapeutic drug for BC patients. As CADD522 decreases Glut-1 expression and glucose consumption, combinatorial treatments with glycolysis inhibitors and CADD522 may be warranted to overcome drug resistance. Collectively, it would be an innovative approach to include CADD522 in combination with standard therapy to improve disease free survival.

## MATERIALS AND METHODS

### Cell culture, reagents and transfection

All breast cancer cell lines were obtained from American Type Culture Collection. Cells were subjected to routine cell line quality examinations (*e.g.,* morphology, Mycoplasma) every 6 months. The cells for experiments were passaged for less than 6 months. Cell lines were maintained in DMEM/F12 (50:50) (BT474 and MDA-MB-468), RPMI1640 (HCC1937, HCC1428, BT549, HCC70), McCoy-5A (SKBr3), and DMEM (the other BC cancer cells) supplemented with 10% FBS, 100 U/mL penicillin and 100 mg/mL streptomycin. Early passage (<10) of IEC-6 undifferentiated rat intestinal crypts and GES-1 immortalized human gastric mucosal cells were provided by Dr. JY Wang (University of Maryland) and Dr. D Kidane (University of Texas) respectively. IEC-6 and GES-1 were grown in DMEM supplemented with 5% FBS, Insulin (1 μg/ml) and Gentamycin (5 μg/ml) and in RPMI1640, respectively. HBME-1 human bone marrow-derived endothelial cells and C2C12 murine myoblast cells were grown in DMEM. MCF10A immortalized human breast epithelial cells were grown in MCF10A-specified media (DMEM/Ham’s F-12 supplemented with 100 ng/ml cholera toxin, 20 ng/ml epidermal growth factor (EGF), 0.01 mg/ml insulin, 500 ng/ml hydrocortisone, and 5% chelex-treated horse serum). All of the growth factors were purchased from Sigma (St. Louis, MO).

Establishment of MCF7-tet-off BC cells with inducible RUNX2 expression was previously described [17], and doxycycline was removed for 72 hrs to achieve maximal RUNX2 protein levels. T47D-RUNX2 and MCF7-RUNX2 cells stably expressing ectopic RUNX2 were cloned after T47D and MCF7 cells were transfected with pCMV.Tag2-RUNX2 expression plasmid (Stratagene) and selected under G418 (0.5 mg/ml). T47D-Empty and MCF7-Empty were cloned in parallel. Hs578t-non-targeting shRNA control (NTC) and -RUNX2 knockdown (KD) cells were cloned under puromycin (2 μg/ml) selection as described [17]. Both MCF7 and T47D cells express relatively low levels of endogenous RUNX2 compared to Hs578t and MDA-MB-231 [16], and MDA-MB-468 cells express moderate levels of RUNX2 compared to MDA-MB-231. CADD522 was purchased from ChemBridge Corporation (San Diego, CA). Doxycycline, Cycloheximide and MG132 were purchased from Sigma/Aldrich (St Louis, MO).

MDA-MB-231-Luc-Hyg (231-Luc) and MCF7-tet-off-Luc-Puro cells stably expressing firefly luciferase (Luc) were cloned under hygromycin (250 μg/ml) and puromycin (0.5 μg/ml) selection, respectively. The bioluminescence intensities in 231-Luc and MCF7-tet-off-Luc (-Doxy) were over 600 and 900-fold higher than those in parental MDA-231 and MCF7-tet-off (-Doxy), respectively (Supplementary Figure 8G), indicating that the bioluminescence intensity of these cells was sufficient for *in vivo* bioluminescence imaging analysis.

Small-Interfering RNA (siRNA) pool targeting RUNX1, RUNX2, and non-targeting control were purchased from Dharmacon, and transfected into cells using RNAiMAX Reagent (Invitrogen). Western blot analysis was performed 48 hrs after transfection.

### DNA-binding enzyme-linked immunosorbent assay (D-ELISA)

A microtiter plate-based D-ELISA was performed as described [43, 44]. Briefly, nuclear proteins were isolated from HBME-1 cells that express all three RUNX proteins (Supplementary Figure 7A) and bound to double-stranded DNA oligonucleotides of human osteocalcin [43, 44] and MMP13 (Forward, 5’- TTC TAC CAC AAA CCA CAC TCG TTC TAC CAC AAA CCA CAC TCG TTC TAC CAC AAA CCA CAC TCG-Biotin -3’ and reverse, 5’- CGA GTG TGG TTT GTG GTA GAA CGA GTG TGG TTT GTG GTA GAA CGA GTG TGG TTT GTG GTA GAA-Biotin -3’). Vehicle (0.05% DMSO) or CADD522 at different concentrations were incubated with the proteins and DNA oligonucleotide mixture in Avidin-coated 96-well plates. DNA-bound RUNX proteins were captured with specific antibodies (Cell Signaling Technologies). Primary and secondary antibody dilution was 1:500 and 1:10,000, respectively.

### Chromatin immunoprecipitation (ChIP) assay

ChIP was performed using a kit (Cell Signaling Technologies) as per manufacturer’s instructions. Briefly, cells were crosslinked with 1% formaldehyde and 1.5 mmol/L ethylene glycol bis[succinimidylsuccinate] at room temperature. Crosslinked chromatin was subsequently harvested, sheared, and precipitated with RUNX2 antibody or nonspecific IgG control (Cell Signaling Technologies). Precipitated DNA was treated with proteinase K, purified and processed for PCR, and amplified PCR products (99 bp) were visualized in 4% agarose gel. Fold enrichment of precipitated DNA over input chromatin was determined in triplicate by quantitative-PCR. PCR primers were designed to amplify regions on the MMP13 proximal promoter region adjacent to the TSS where the Runt binding element resides. Forward, 5’- GGT TTT GAG ACC CTG CTG AA-3’ (-229 bp ∼ -209 bp) and Reverse 5’-CGT GGC GAC TTT TTC TTT TC-3’ (-150 bp ∼ -131 bp).

### Cell growth assay

Cells wereplated on 96-well (30,000 cells/well) or 24-well plates (50,000 cells/well). After CADD522 (0 ∼ 100 μM) addition, cells were incubated for 24 ∼ 72 hrs. Cells were stained with crystal violet (0.5% in Methanol:Acetic Acid=3:1) and washed with PBS. Crystal violet was solubilized in DMSO and measured in a microplate reader at 592 nm.

### Cell cycle analysis

Cells (1 x 10^6^ cells/ml) were starved in serum-free medium for 24 hrs, and released in 10% serum-medium with or without CADD522 (50 μM) for 16∼24 hrs for cell cycle transition. Cells were then fixed in 70% ethanol at 4°C for 1 hr, resuspended in 1 ml PBS containing 20 μg/ml propidium iodide, 20 μg/ml RNase A, incubated for 30 min at room temperature, and analyzed with a Becton Dickinson LSR-II at the Flow Cytometry Core Laboratory at the University of Maryland. Ten thousand events per sample were collected and analyzed using the Cell-Quest (BD Biosciences).

### Caspase 3/7 assay

Cells were cultured in 96-well plates (30,000 cells/well). After CADD522 treatment for 24∼72 hrs, cellular apoptosis was analyzed using the Caspase-Glo 3/7 Assay kit (Promega) according to the manufacturer’s instructions.

### Clonogenic survival assay

Cells were plated on 6-well plates (200 ∼ 500 cells/well). After CADD522 (50 μM) treatment, cells were incubated for 2∼3 weeks without changing media. Colonies were fixed in Methanol-Acetic Acid solution (3:1) and stained with crystal violet (0.5%). After washing, colonies were photographed and counted.

### Anchorage-independent cell growth assay

Cells (10,000) were mixed in 1 ml of 0.3% low-melting agarose over a 0.6% agar bottom layer in normal growth media. The medium (600 μl) with or without CADD522 (50 μM) on soft agar was changed three times a week for 2∼3 weeks. Images of MDA-231 and MCF7 were taken using Nikon Eclipse TE-2000S microscope with Zen Pro image software, and of MDA-468 using Olympus CKX41 microscope with Q-Capture Pro 7 image software at indicated magnifications under the same exposure settings for corresponding vehicle and drug treatments.

### Tumorsphere assay

Single cell suspensions (100,000 cells/well) wereplatedin 6-well ultra-low attachment plates (Corning) with 5 ml of EGM-2 supplemented with bullet kit (Lonza) and 2% FBS. CADD522 (50 μM) was added at the day of the plating or 4 days after plating. Tumorspheres were continuously photographed for 18 days and counted at the final day. Spheres were counted at 9 fields per well and averaged from triplicate.

### Cell invasion assay

MCF7-tet-off cells were grown in the media with (+Doxy) or without doxycycline (–Dox) for 72 hours. Cells were then treated with CADD522 (50 μM) for 24 hrs, and trypsinized and suspended in serum-free media. Cells were re-plated in the top chamber precoated with 0.1X BME (Cultrex), and growth medium supplemented with 10% FBS was used as a chemoattractant in the bottom chamber. CADD522 was added to both chambers and incubated for 16 hrs. Cellular invasion was analyzed according to the 96 Well BME Cell Invasion Assay protocol (Cultrex).

### Cell-electrode impedance invasion assay (xCELLigence System)

Real-time monitoring of cellular invasion was examined using an electrical impedance assay with an xCELLigence RTCA SP real-time cell-sensing device (Roche Applied Science). Matrigel (20 μl of 0.5 mg/ml) was pre-coated in the upper chamber of CIM plates and polymerized for 4 h and MDA-231 cells (75,000) were seeded onto wells containing growth medium with vehicle (0.1% DMSO) or CADD522 (50 μM). Impedance-based signals were measured every 5 minutes for 48 hrs according to the manufacturer’s instructions. The invasive activity is expressed as the cell index (mean ± SD) of duplicate wells. Three independent experiments were performed. In parallel, MDA-231 cells were plated onto wells with serum-free medium and the assay was performed.

### Luciferase-promoter reporter assays

Cells were plated in 96-well plates (30,000 cells/well) and incubated overnight. Cells were co-transfected with indicated luciferase-reporter plasmids (25 ng/well) and pSV-Renilla-Luc (Promega) (15 ng/well) for 6 hrs and CADD522 (0 ∼ 100 mM) was further treated for 18∼48 hrs. COX-2 (P2-274)-Luc (-170bp∼+104bp) and control plasmids (PXP2-Luc) were kindly provided byDr. Miguel A. Iñiguez (Universidad Autónoma de Madrid, Spain), and pNF-kb-Luc was from Dr. Hancai Dan (University of Maryland). Luciferase assay was performed using the Dual-Glo Luciferase Assay Systems as per manufacturer’s recommendation (Promega).

### Quantitative real time-RT-PCR (Q-RT-PCR)

Total RNA was extracted using TRIzol (Life Technologies). One μg of total RNA was reverse transcribed with oligo-(dT) primer using the SuperScript first-strand synthesis system (Invitrogen) to synthesize cDNA. One μl of each cDNA was used for real-time RT-PCR using QuantiFast SYBR Green PCR Kit (Promega). mRNA expression of gene of interest relative to β-actin was calculated based on the threshold cycle (C_t_) as 2^-^D(DCt) method. Primer sequences are listed in Supplementary Table 1.

### Western blot analysis

Whole cell lysates extracted in RIPA buffer (Upstate) were separated on 4–12% gradient SDS-PAGE and transferred to nitrocellulose membrane. The blots were incubated with specific antibodies for each protein overnight at 4°C. After antibody washing, the blots were reacted with their respective secondary antibody and detected with enhanced chemiluminescence reagents (Millipore) according to the supplier’s protocol. Antibodies for proteins were purchased as below. RUNX1, RUNX2, CBF-β, ubiquitin and caspase-3 (Cell Signaling Technologies), S451-p-RUNX2 (Bioss Gentaur, Belgium), RUNX3 and Glut-1 (Millipore, CA), β-actin and RUNX2 antibody for IHC (Sigma-Aldrich), and Ki-67 for IHC (Bethyl Laboratories, Inc, TX).

### Glucose consumption and lactate production

For measurement of glucose level, cells (100,000/well) were plated in 24-well plates and incubated overnight. Cells were treated with CADD522 (50 μM) in phenol red-free growth medium and further incubated for 6 hrs or 24 hrs. The culture medium was collected and filtered through a 0.22 μm pore membrane. Glucose level in the medium was measured using Amplex Red Glucose/Glucose Oxidase Assay Kit (Invitrogen) as per manufacturer’s instructions. For lactate level, cells were treated with CADD522 for 24 hrs, and the medium was washed and replaced with HEPES-buffered Krebs-Ringer solution (Boston BioProducts) supplemented with 10 mM Glucose. Cells were incubated for 30 min, and the medium was collected and filtered through a 0.22 μm pore membrane. Lactate level in the medium was measured using Lactate Assay Kit (Sigma-Aldrich) as per manufacturer’s instructions. Cell growth assays using crystal violet staining were performed to verify equal number of cells and no significant difference was found in 24 hrs of CADD522 treatment (data not shown).

### *In vivo* animal studies and immunohistochemical (IHC) analysis

Animal maintenance and experimental protocols are in accordance with the guidelines of the University of Maryland’s Institutional Animal Care and Use Committee. Results from the maximum tolerated dose (MTD) test performed prior to the *in vivo* study showed that doses of CADD522 up to 20 mg/kg mice for 2 weeks in athymic nude mice had no detectable influence on body weight or the general health of animals (data not shown).

For the MMTV polyoma middle T antigen (PyMT) mouse model, female mice were purchased from Jackson Laboratory. As mice developed first palpable mammary tumors after 5 weeks of age, mice were randomly assigned to 4 treatment groups at 6 weeks of age and received *i.p.* injections of CADD522 (1, 5 and 20 mg/kg/group) or equivalent volumes of vehicle (10% DMSO/90% PBS) (200 μl) twice a week. Palpable tumors were monitored every 1 to 2 days untll 12 weeks of age to determine tumor incidence (number of tumors per mouse) and onset (the age of palpable tumors). Tumor weight was quantified at the final day after mice were euthanized and tumors were excised. Tumor volume [(length × width^2^)/2] was measured by caliper.

For the PDX models, Br-001 tumor fragments derived from a patient with TNBC (University of Maryland, Translational Core Facility) were inoculated subcutaneously in female NOD *scid* gamma (NSG) mice (P1). After P1 tumors were grown, tumors were excised, fragmented and inoculated to a new group of NSG mice (P2). After P2 tumors were grown, they were excised, fragmented, and inoculated into nude mice (P3). When the size of the tumors reached 200 ∼ 250 mm^3^, mice were randomized into two groups, and received *i.p.* injection of vehicle or CADD522 (10 mg/kg) twice a week for 11 days.

To compare RUNX2 expression, protein lysates of normal mammary gland and mammary tumor samples were isolated from age-matched wild-type and MMTV-PyMT transgenic mice, respectively. Tumor tissues were dissected and processed for preparation of FFPE sections. After hematoxylin and eosin (H&E) staining was performed on each sample, IHC analysis was performed with specific antibodies recognizing RUNX2, Ki-67 (proliferation), caspase-3 (apoptosis), and Vector ABC kit (avidin-biotin-HRP) was used for detection.

For *in vivo* lung metastasis assay, MCF7-tet-off-Luc cells were grown for 3 days in the absence of Doxycyclin (-Doxy) and injected into the tail vein of the 8 week-old female NSG mice (1 x 10^6^ cells/200 μl of PBS). After inoculation, the mice were randomly assigned to vehicle control and treatment groups. Vehicle (10% DMSO/90% PBS) and CADD522 (10 mg/kg) were injected *i.p*. 2 hrs after inoculation. The retention of the cells in thelung was monitored by noninvasive bioluminescence imaging for 24 hrs. For luciferase detection, 150 mg/mL D-luciferin (Caliper Life Sciences) in PBS was injected *i.p.* before imaging. Photometric measurement of metastasis was done by living Image software (Xenogen). In separate groups of mice, 231-Luc cells (1 x 10^6^/200 μl of PBS) were injected, and the metastasis burden in the lung was quantified for 3weeks.

### Statistical analysis

Results from cell culture assays were expressed as the mean ± SD from at least three independent experiments. Comparisons of quantitative data between two groups were analyzed using the two-tailed Student’s *t-test*. For *in vivo* study, data were expressed as the mean ± SE. Multiple comparisons were followed by Mann-Whitney non parametrical tests. All statistical analyses were conducted using STATA version 14 (STATA Inc., College Station, TX). P values less than 0.05 were considered significant.

## SUPPLEMENTARY MATERIALS FIGURES AND TABLE





## Abbreviations

BC: breast cancer
TNBC: triple-negative breast cancer
RUNX2: runt-box domain protein-2; transcription factor
OCR: oxygen consumption rate
PDH: pyruvate dehydrogenase
CADD: Computer-Assisted Drug Design
D-ELISA: DNA-binding ELISA
OSE2: osteoblast-specific element 2
OC: osteocalcin
CHX: cycloheximide
PDX: patient-derived xenograft
CBF: core binding factor
