# Supplementary material for: Characterization of CADD522, a small molecule that inhibits RUNX2-DNA binding and exhibits antitumor activity
Source: Oncotarget. 2017 Aug 10;8(41):70916–40. doi: 10.18632/oncotarget.20200 (PMC5642608; doi:10.18632/oncotarget.20200)
Supplement: Supplementary file 1 [file oncotarget-08-70916-s001.pdf]

Characterization of CADD522, a small molecule that inhibits RUNX2-DNA binding and exhibits antitumor activity

SUPPLEMENTARY MATERIALS

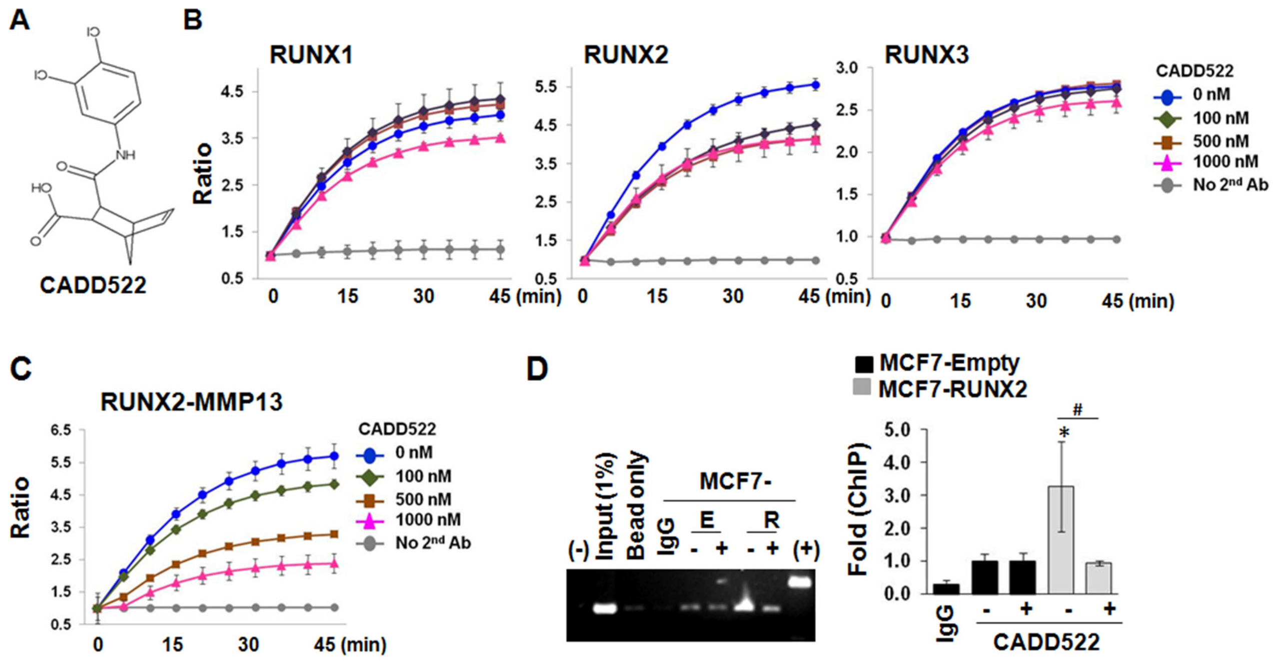

Supplementary Figure 1: (A) Chemical structure of CADD522 (C15H13Cl2NO3, MW of 326.175). **(B)** D-ELISA was performed using nuclear proteins from HBME-1 and OSE2 oligonucleotides as reported [43, 44]. Ten µg of nuclear proteins in 100 µl reaction volume was incubated with specific antibodies at 4ºC overnight. Absorbance at 635 nm was measured every 5 min (kinetic program). All determinations were in triplicate and shown as mean ± SD. Blue circle, 0 nM CADD522; dark green diamond, 100 nM; brown square, 500 nM; pink triangle, 1000 nM; grey circle, no secondary antibody. X-axis, 5 min interval for 45 min. Ratio, Relative value to value at 0 min. 0.05% DMSO was used as vehicle control (0 nM of CADD522). **(C)** D-ELISA was performed using the MMP13 oligonucleotides that harbor the Runt binding sequence and RUNX2 antibody. **(D)** MCF7-Empty and -RUNX2 cells were treated with CADD522 (50 µM) for 24 hrs, and MMP13 ChIP analysis was performed with RUNX2 or IgG antibody. Eluted gDNA was processed for conventional- (left) and quantitative-PCR (right). (-), no template; (+), ChIP positive control (RPL-30 provided by the kit). Fold (ChIP) was calculated from the ratio to MCF7-Empty without CADD522 after normalization to Input (1%) (mean ± SD). Experiments were done in triplicate and repeated twice. *, *P<0.05* compared to MCF7-Empty without CADD522 treatment. #, *P<0.05* compared to MCF7-RUNX2 without CADD522 treatment.

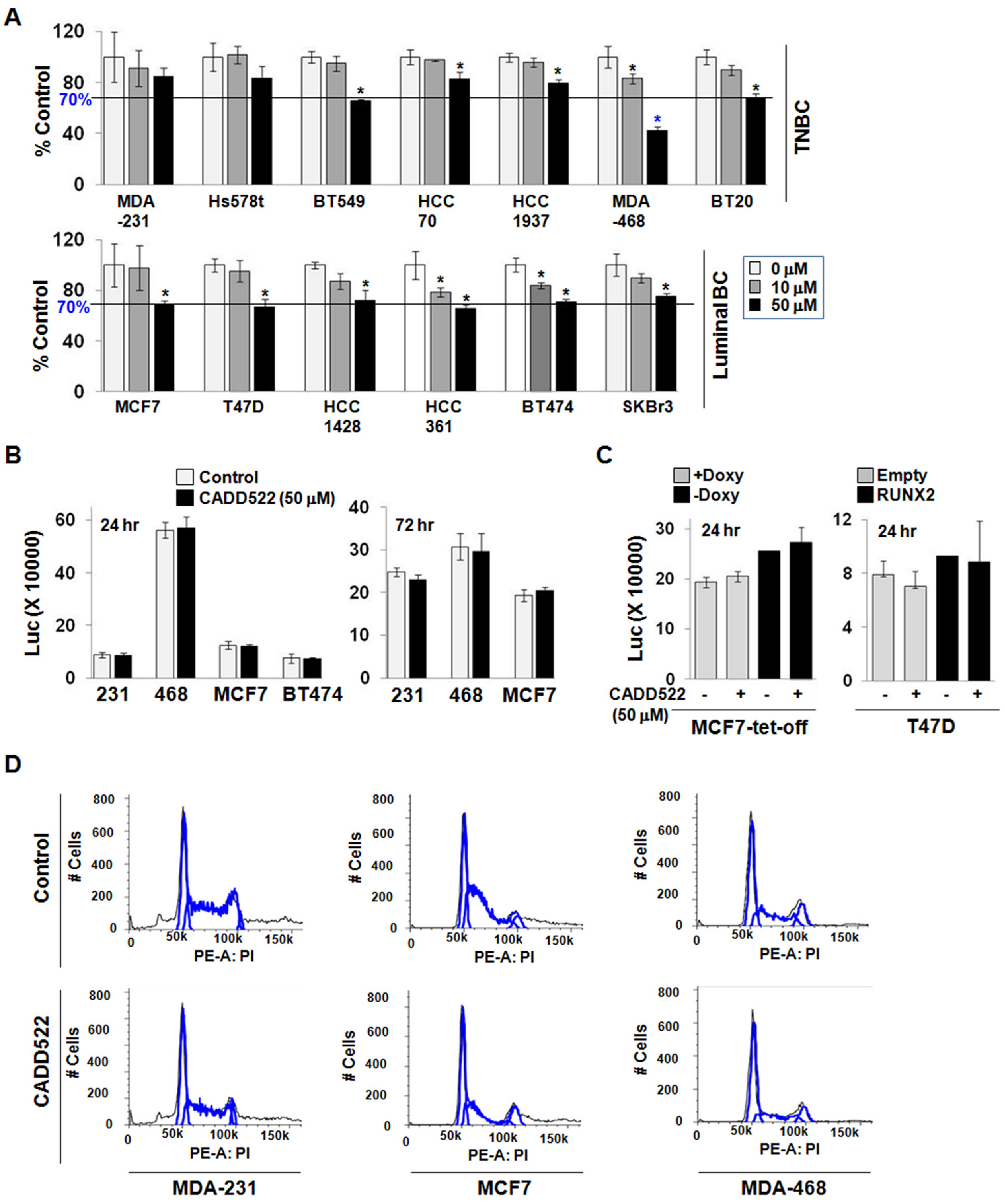

Supplementary Figure 2: (A) Cell growth assay in TNBC and luminal-type BC cell lines. *, *P<0.05* compared to vehicle controls. **(B)**, MDA-231, MDA-468 and MCF7 cells were treated with CADD522 for 24 hrs or 72 hrs, and cellular apoptosis was determined by the Caspase-3/7 assay. 231, MDA-231; 468, MDA-468. **(C)** MCF7-tet-off cells with or without doxy removal and T47D-Empty and T47D-RUNX2 cells were treated with CADD522 for 24 hrs, and cellular apoptosis was determined by the Caspase-3/7 assay. Data are presented as luminescence intensity (Luc X 10,000). -, Vehicle controls; +, CADD522-treated cells. **(D)** Images of cell cycle analysis of Figure 1D were shown. # cells, number of cell count.

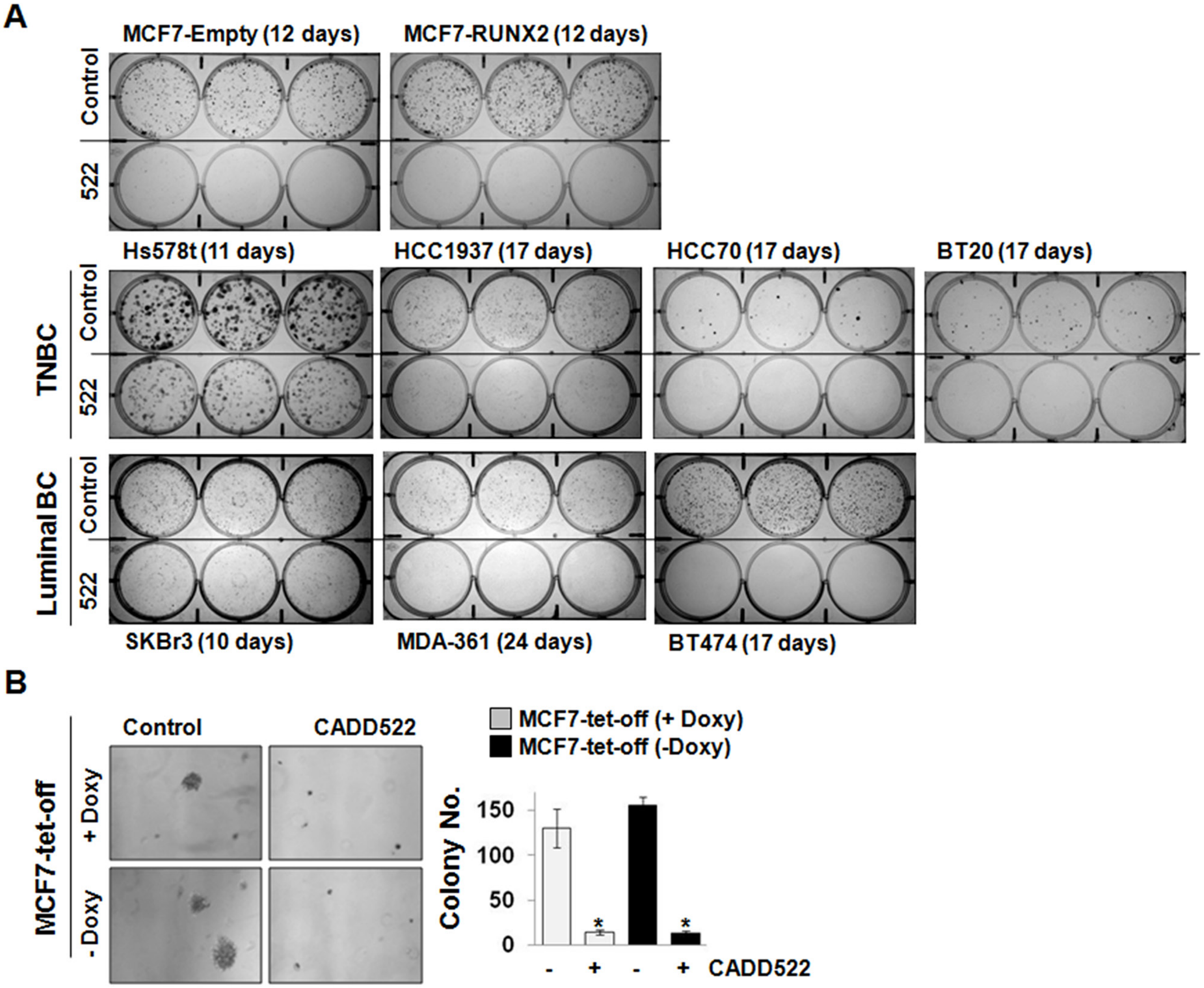

Supplementary Figure 3: Clonogenic assay was performed to determine cell survival of various BC cell lines after CADD522 treatment (50 μM) for 2 ~ 3 weeks, and colonies were photographed. The periods of CADD522 treatment are indicated for each cell line. **(A)** Clonogenic assay was performed to determine cell survival of various BC cell lines after CADD522 treatment (50 μM) for 2 ~ 3 weeks, and colonies were photographed. The periods of CADD522 treatment are indicated for each cell line. **(B)** Anchorage-independent cell growth of MCF7-tet-off cells with or without Doxy removal.

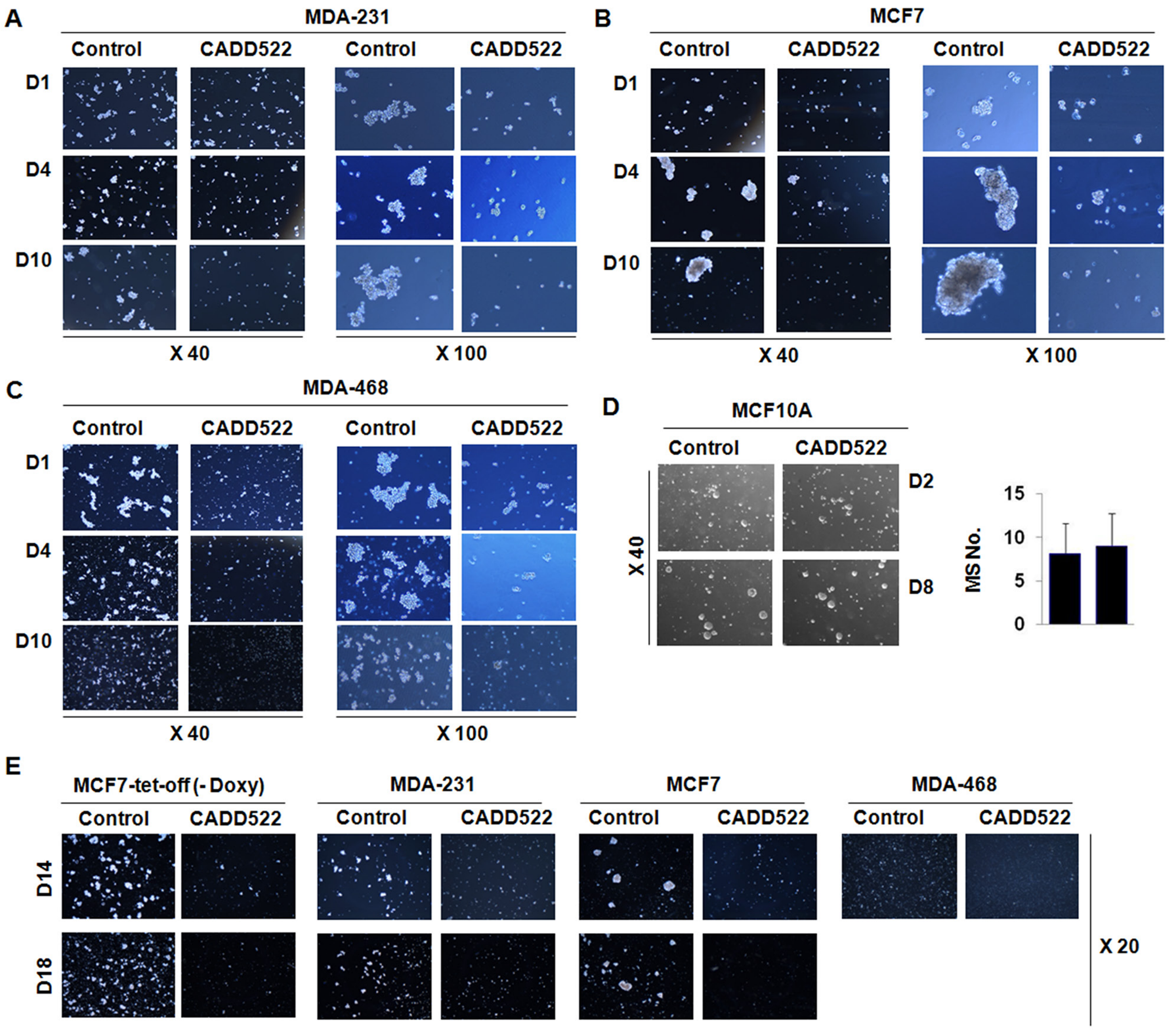

Supplementary Figure 4: Tumorsphere formation of BC cells within 10 days **(A-C)** and after 14 days **(E). (D)** Mammosphere photos of MCF10A. CADD522 (50 µM) was added at day 0 (D0, the day of plating cells) and cells were incubated for 8 days.

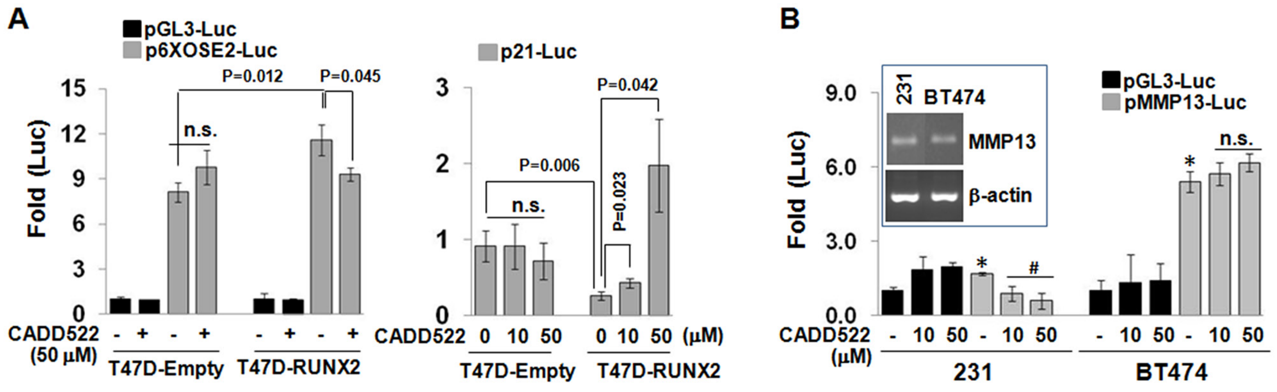

Supplementary Figure 5: (A) T47D-RUNX2 and T47D-Empty cells were transfected with the indicated luciferase plasmids and treated with CADD522 for 48 hrs. Relative Luc activity (Fold) was calculated from the ratio of indicated luciferase plasmids activity to pGL3 activity after normalization of pRenilla activity. Data presented as mean ± SD. Experiments were done in triplicate and repeated twice. *P* values are indicated, and *<0.05* were considered significant. **(B)** MDA-231 cells that express both RUNX1 and RUNX2 andBT474 cells that express only RUNX1 among RUNX family proteins were transfected with pMMP13-Luc plasmids and treated with CADD522 for 48 hrs. *, *P<0.05* compared to pGL3-Luc with vehicle control. #, *P<0.05* compared to pMMP13-Luc with vehicle control. n.s., not significant. Box, MMP13 mRNA expression in BT474 cells was confirmed by RT-PCR analysis. MDA-231 cells were used for a positive control.

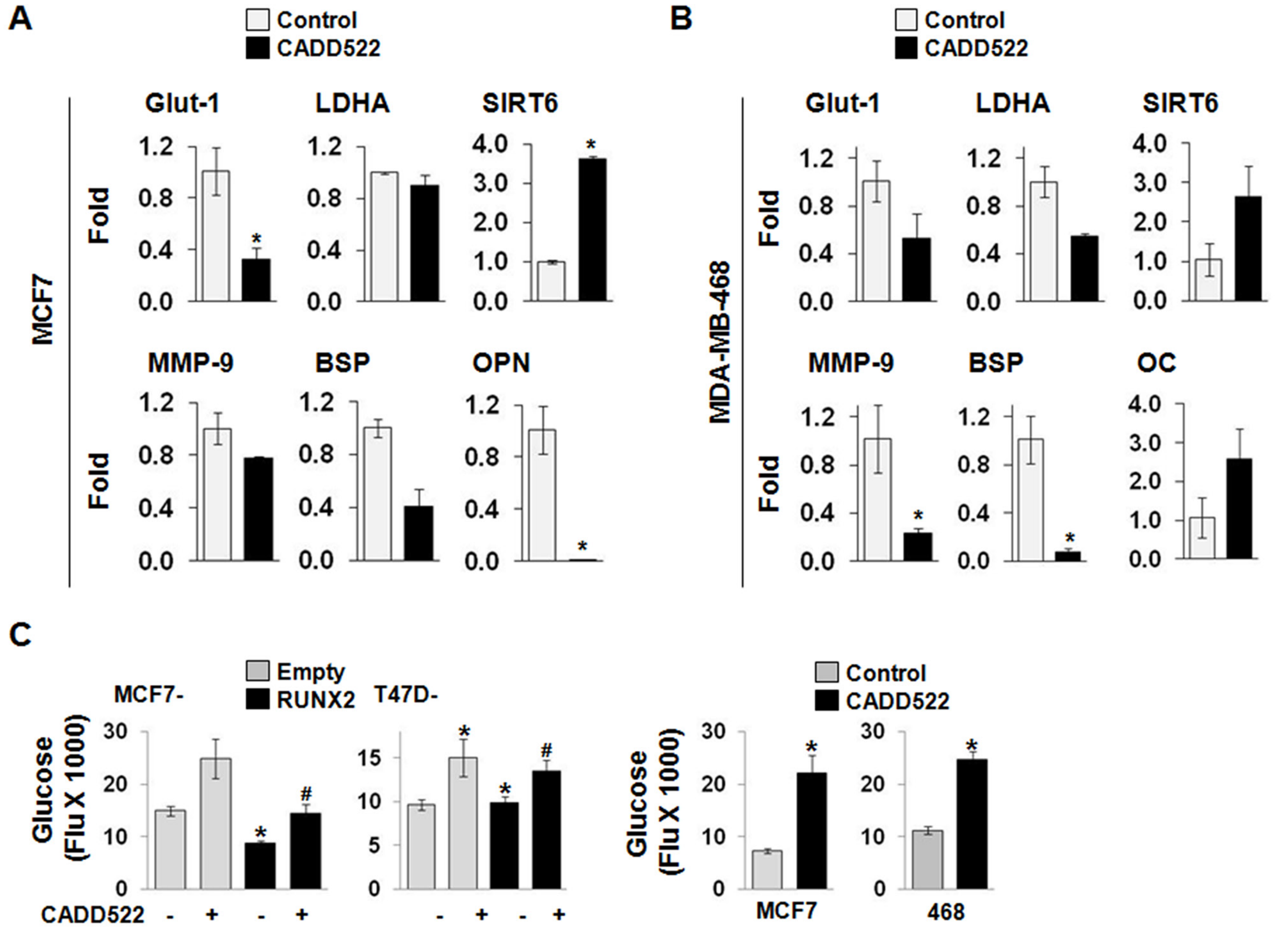

Supplementary Figure 6: Q-RT-PCR analyses of RUNX2 target genes in MCF7 **(A)** and MDA-468 cells **(B)**. Cells were treated with or without CADD522 (50 µM) for 6 hrs. **(C)** The levels of glucose in the cell culture medium prepared from BC cells treated with or without CADD522 for 24 hrs.

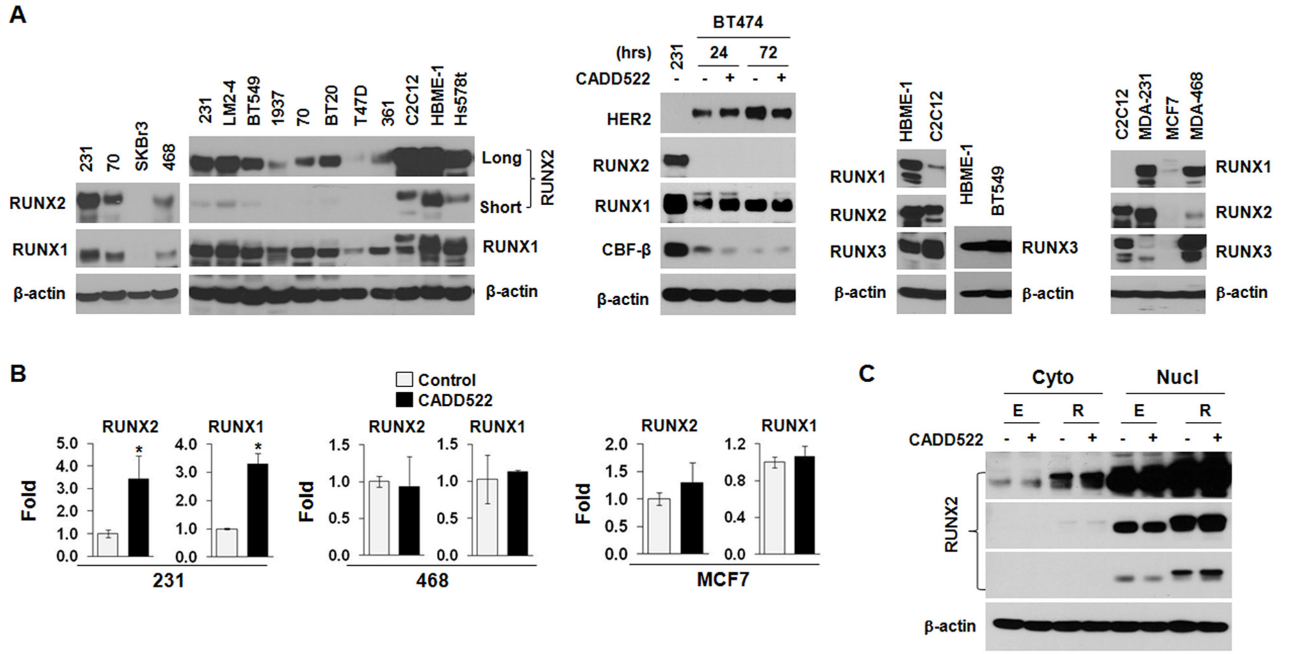

Supplementary Figure 7: (A) Expression of RUNX proteins in BC cell lines. Ubiquitous expression of RUNX1 and RUNX2 2 in BC cell lines. RUNX2 in T47D and HCC1428 cells were reported previously [16]. HBME-1, C2C12 and BT549 were positive for all three RUNX proteins. HCC70, MDA-468, BT20 and MDA-MB-361 cells were reported to be RUNX3-positive whereas the other BC cell lines were RUNX3-negative [30]. SKBr3 cells were negative for all RUNX proteins. C2C12, a mouse myoblast cell line. Dark and light images of RUNX2 expression from long and short exposure, respectively, are shown. BT474 cells do not express RUNX2, and the CBF-β level was reduced with CADD522 treatment for 24 hrs. **(B)** Q-RT-PCR analysis in MDA-231, MDA-468 and MCF7 cells treated with CADD522 for 24 hrs. *, *P<0.05* compared to vehicle controls. **(C)** MCF7-Empty and -RUNX2 cells were treated with CADD522 (50 µM) for 72 hrs, and RUNX2 level in cytosolic (Cyto) and nuclear (Nucl) fraction was determined. Upper, middle and lower panels of RUNX2 were from relatively long and short film exposure of the same membrane.

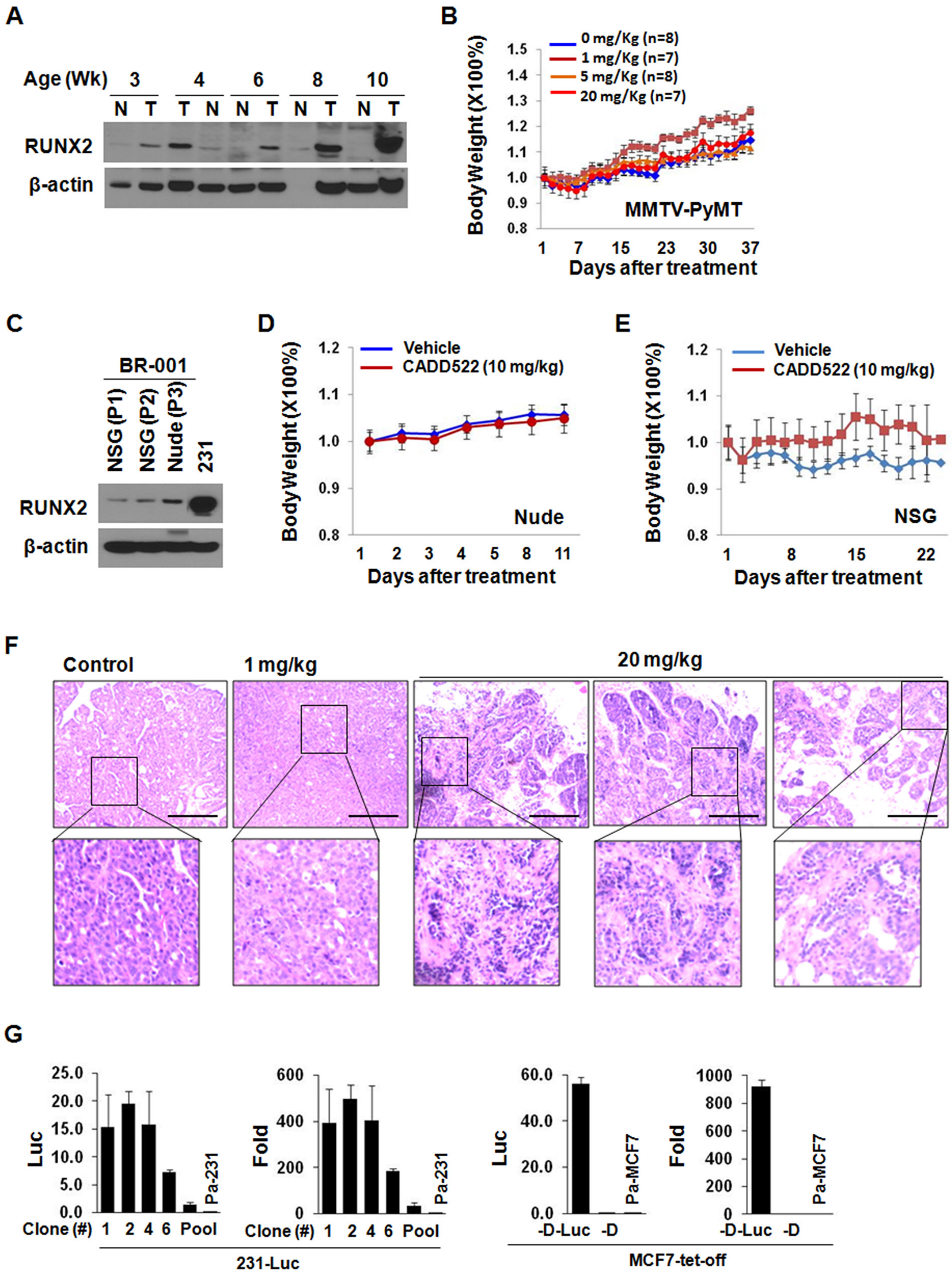

Supplementary Figure 8: (A) Normal mammary gland (N) and mammary tumor samples (T) isolated from age-matched wild-type or MMTV-PyMT transgenic mice were analyzed for RUNX2 expression. Wk, week. **(B)** No significant decrease on body weight was observed in CADD522-injected MMTV-PyMT mice. Values in Y-axis, % body weight from day 1. **(C)** RUNX2 expression in consecutive passages of the TNBC-PDX Br-001 model. Protein lysate from MDA-231 cells was used as a positive control. Percentage of body weight from day 1 in TNBC-PDX Br-001 bearing mice **(D)** and 231-luc injected NGS mice **(E)**. **(F)** H&E staining of representative tumors from MMTV-PyMT mice. CADD522-treated tumors retained glandular morphology. Scale bar, 200 mm. **(G)** MDA-MB-231-Luc-Hyg (231-Luc) and MCF7-tet-off-Luc-Puro cells stably expressing firefly luciferase (Luc) were cloned under hygromycin (250 µg/ml) and puromycin (0.5 µg/ml) selection, respectively as described in Materials and Methods. Luciferase activity (Luc, bioluminescence intensity/mg protein/1000) in 231-Luc and MCF7-tet-off-Luc (-Doxy) cells (-D-Luc) were determined, and relative (Fold) was calculated by the luciferase activity of stable clones vs. that of parental, non-transfectants (Pa). –D, MCF7-tet-off (-Doxy) cells. Experiments were done in triplicate, and data are presented as mean ± SD.

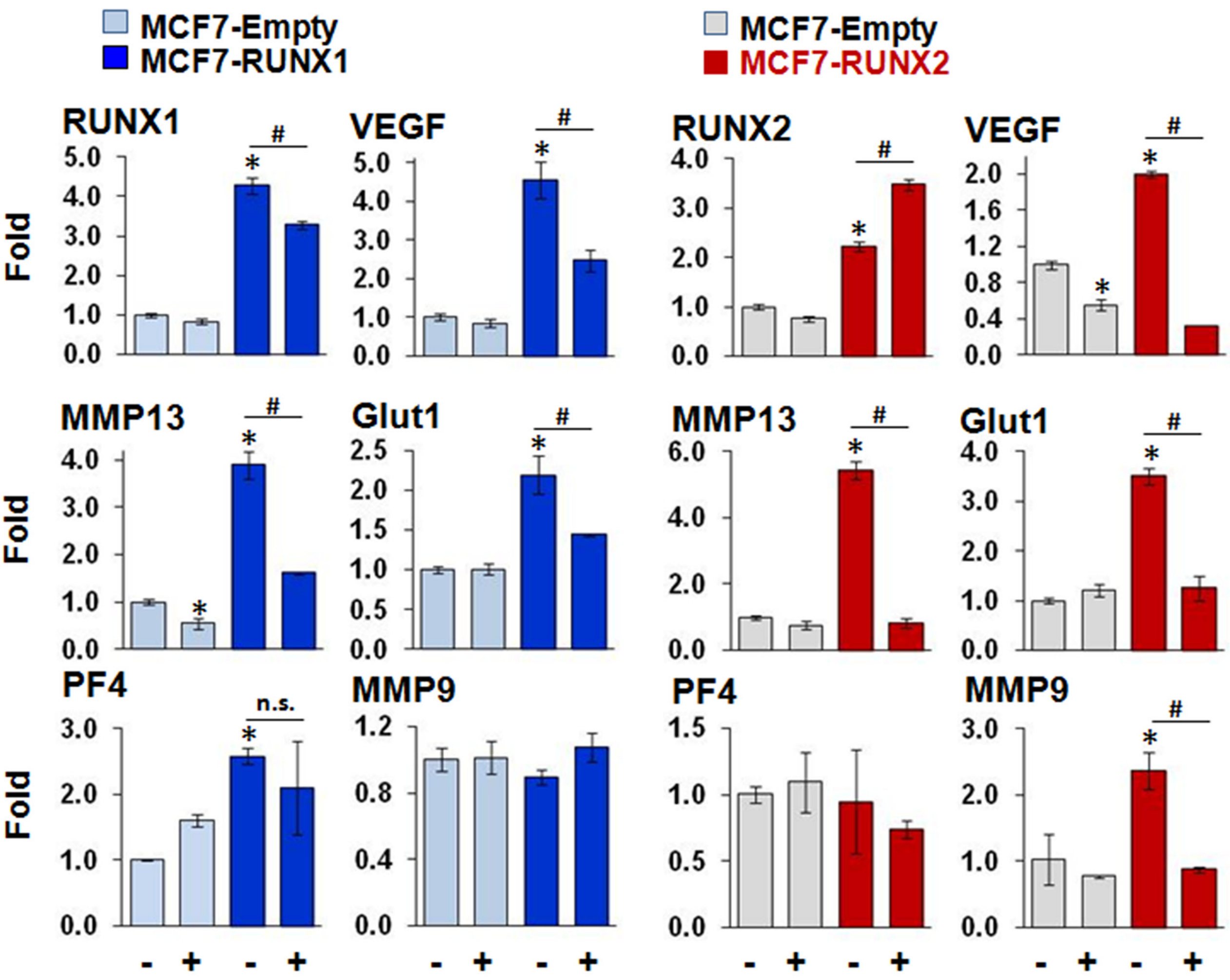

Supplementary Figure 9: MCF7 cells overexpressing RUNX1 (MCF7-RUNX1) or RUNX2 (MCF7-RUNX2) and control cells (MCF7-Empty) were treated with CADD522 for 72 hrs, and Q-RT-PCR analysis was performed. Gene expression levels relative to 18S rRNA level were normalized to vehicle-treated cells. -, vehicle (0.1% DMSO); +, CADD522 (50 mM). Ectopic RUNX1 was expressed in MCF7 cells by transient transfection, and RUNX2 was stably expressed. n.s., not significant.

Supplementary Table 1: Primers for Q-RT-PCR

See Supplementary File 1
